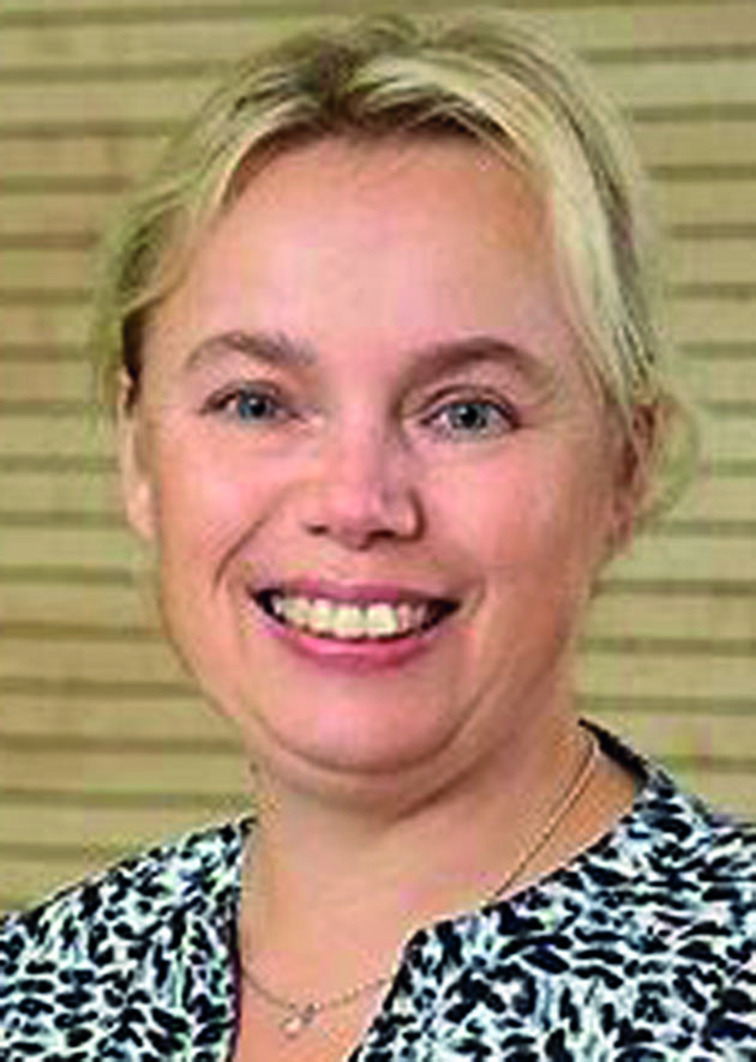# Life Cycle of LiFePO_4_ Batteries: Production, Recycling, and Market Trends

**DOI:** 10.1002/cphc.202400459

**Published:** 2024-11-03

**Authors:** Hossein Rostami, Johanna Valio, Pekka Tynjälä, Ulla Lassi, Pekka Suominen

**Affiliations:** ^1^ University of Oulu Research Unit of Sustainable Chemistry P.O.Box 3000 FI-90014 Oulu Finland; ^2^ Satakunta University of Applied Sciences (SAMK) Satakunnankatu 23 FI-28130 Pori Finland; ^3^ University of Jyvaskyla Kokkola University Consortium Chydenius Talonpojankatu 2B FI-67100 Kokkola Finland

**Keywords:** Lithium iron phosphate, Spent-batteries, LiFePO_4_ Recycling, Sustainable recovery, LFP market.

## Abstract

Significant attention has focused on olivine‐structured LiFePO_4_ (LFP) as a promising cathode active material (CAM) for lithium‐ion batteries. This iron‐based compound offers advantages over commonly used Co and Ni due to its lower toxicity abundance, and cost‐effectiveness. Despite its current commercial use in energy storage technology, there remains a need for cost‐effective production methods to create electrochemically active LiFePO_4_. Consequently, there is ongoing interest in developing innovative approaches for LiFePO_4_ production. While LFP batteries exhibit significant thermal stability, cycling performance, and environmental benefits, their growing adoption has increased battery disposal rates. Improper disposal practices for waste LFP batteries result in environmental degradation and the depletion of valuable resources. This review comprehensively examines diverse synthesis approaches for generating LFP powders, encompassing conventional methodologies alongside novel procedures. Furthermore, it conducts an in‐depth assessment of the methodologies employed in recycling waste LFP batteries. Moreover, it emphasizes the importance of LFP cathode recycling and investigates pretreatment techniques to enhance understanding. Additionally, it provides valuable insights into the recycling process of used LFP batteries, aiming to raise awareness regarding the market for retired LFP batteries and advocate for the enduring sustainability of lithium‐ion batteries.

## Graphical abstract

This review investigates various synthesis methods for LiFePO_4_ (LFP) as a cathode material for lithium‐ion batteries, highlighting its advantages over Co and Ni due to lower toxicity and cost. It also explores recycling techniques for waste LFP batteries, emphasizing the need for efficient recycling to prevent environmental harm, conserve resources, and ensure the sustainability of lithium‐ion batteries.

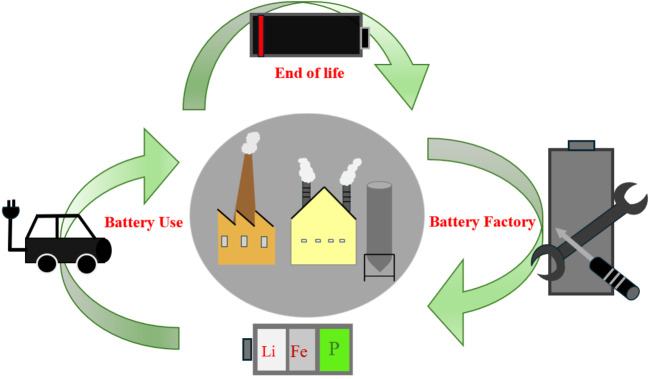

## 1 Introduction

**Figure 1:**
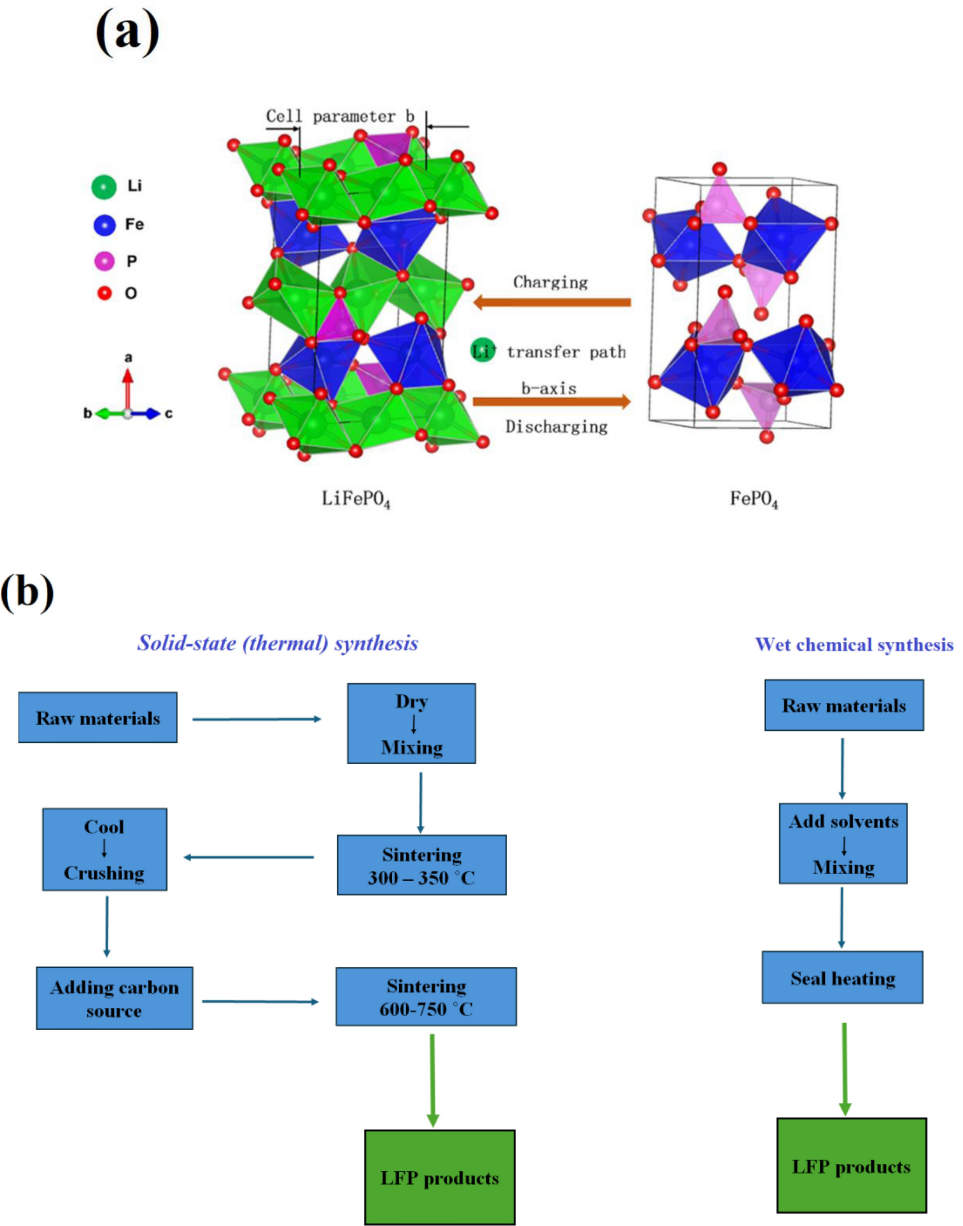
(a) Schematic illustration of the olivine structure of LiFePO_4_, including FeO_6_ octahedra, PO_4_ tetrahedra, and Li^+^ one‐dimensional diffusion channel and the phase transition process. Copyright 2023 Elsevier. Reproduced with permission from reference^[12]^ and (b) Comparison of the process flow between solid and liquid phase method for LFP synthesis.

The olivine structure of LiFePO_4_ (LFP) has a hexagonally close‐packed oxygen array in which the octahedra share both edges and faces (Figure 1a). The arrangement of cations in LiFePO_4_ differs notably from that in layered and spinel structures like LCO and LMO, respectively. Unlike these structures, LiFePO_4_ lacks a continuous network of FeO_6_ edge‐shared octahedra that could enhance electronic conductivity. LiFePO_4_ has emerged as a top positive electrode material in the past decade thanks to a deep understanding of its structural changes during lithium insertion and clever manipulation of particle shapes. Based on this one‐electron process, 170 mAh g^−1^ is the theoretical capacity. However, because of electron and ion transport restrictions, early attempts to remove Li from this material were only able to remove around 0.6 e^−^.^[1]^ The primary hurdle to achieving the theoretical capacity of LiFePO_4_ is its low inherent electronic conductivity. Also, lithium movement within the olivine crystal structure occurs along one‐dimensional channels, with limited opportunities for crossing between these channels. These narrow pathways are especially prone to blockage by defects and impurities.[^2^, ^3^] Several strategies have been investigated to mitigate the conductivity challenge, such as diminishing particle dimensions and achieving uniformity in particle size distribution,^[4]^ coating the particles with carbon,[^5^, ^6^] or synthesizing compounds with carbon to envelop each particle with a proficient electronic conductor,^[7]^ Tailoring the morphology and texture of the particle through low‐temperature synthesis routes,^[8]^ and selective doping with cations to increase the intrinsic conductivity.

The demand for LFP batteries as energy storage devices has significantly increased due to their notable advantages, including long lifespan, enhanced discharge and charge efficiency, and safe usage. Furthermore, the appeal of LFP batteries is further strengthened by the limited availability of critical raw materials for NMC batteries and their cost‐effectiveness, leading to a surge in demand for LFP batteries. The rise in the production of high‐energy storage devices, electric vehicles (EVs), and hybrid electric vehicles (HEVs) has resulted in a significant surge in the utilization of LFP batteries.^[9]^ Indeed, the widespread utilization of LFP batteries elicits dual concerns surrounding their impact on the economy and the environment. The high extraction costs and unpredictable product quality associated with lithium production for LFP synthesis provide economic challenges. The extensive consumption of LFP batteries may lead to a significant issue in the future, as many spent batteries accumulate despite their current operational lifespan.^[10]^ Therefore, recycling these batteries aids in preventing environmental pollution and reclaiming valuable materials for reuse.

This review introduces the current state of recycling for spent LFP batteries and analyzes various recycling technologies. Additionally, it summarizes the latest advancements in novel methods developed in recent years. Up until now, the recycling of spent LFP batteries has mainly been carried out using two traditional methods: (1) pyrometallurgy (i. e., direct regeneration) and (2) hydrometallurgy (i. e., the leaching of individual metals). This review outlines the procedures for synthesizing (doped and coated) LFP powders, starting from the necessary raw materials. Alongside established traditional synthesis routes for LFP, it discusses various alternative methods. It also examines the properties of LFP and introduces essential raw materials for LFP within the battery value chain. Also, the review addresses the limited coverage of recycling technologies for spent LFP batteries, which often concentrate on conventional methods or singular recovery approaches. It highlights critical recycling techniques for LFP cathodes and provides an overview of pretreatment technologies to enhance comprehension. Moreover, it offers insights into the development of spent LFP recycling, aiming to draw attention to the retired LFP market and promote the long‐term sustainability of lithium‐ion batteries.

## 2 Synthesis of LFP

Since John Goodenough discovered the olivine phase's electrochemical characteristics in 1997, numerous synthesis routes of the olivine‐type LiFePO_4_ were explored.^[11]^ A general distinction can be made between processes that start in the solid phase and those that commence in the solution phase.

The synthesis of lithium iron phosphate can be achieved through solid‐phase or liquid‐phase methods. Solid phase techniques like high‐temperature reactions, carbothermal reduction, and microwave synthesis are favored for their simplicity and suitability for industrial production. Lithium iron phosphate is coated with pyrolytic carbon to enhance conductivity in the carbothermal reduction method. Liquid phase methods such as precipitation, sol‐gel, and hydrothermal synthesis, offer uniform mixing and better product consistency but require high‐pressure, high‐temperature conditions, and complex equipment, making mass production challenging. Figure 1b compares the solid‐phase and liquid‐phase synthesis methods.

### 2.1 Solid‐State (Thermal) Synthesis of LFP

**Figure 2:**
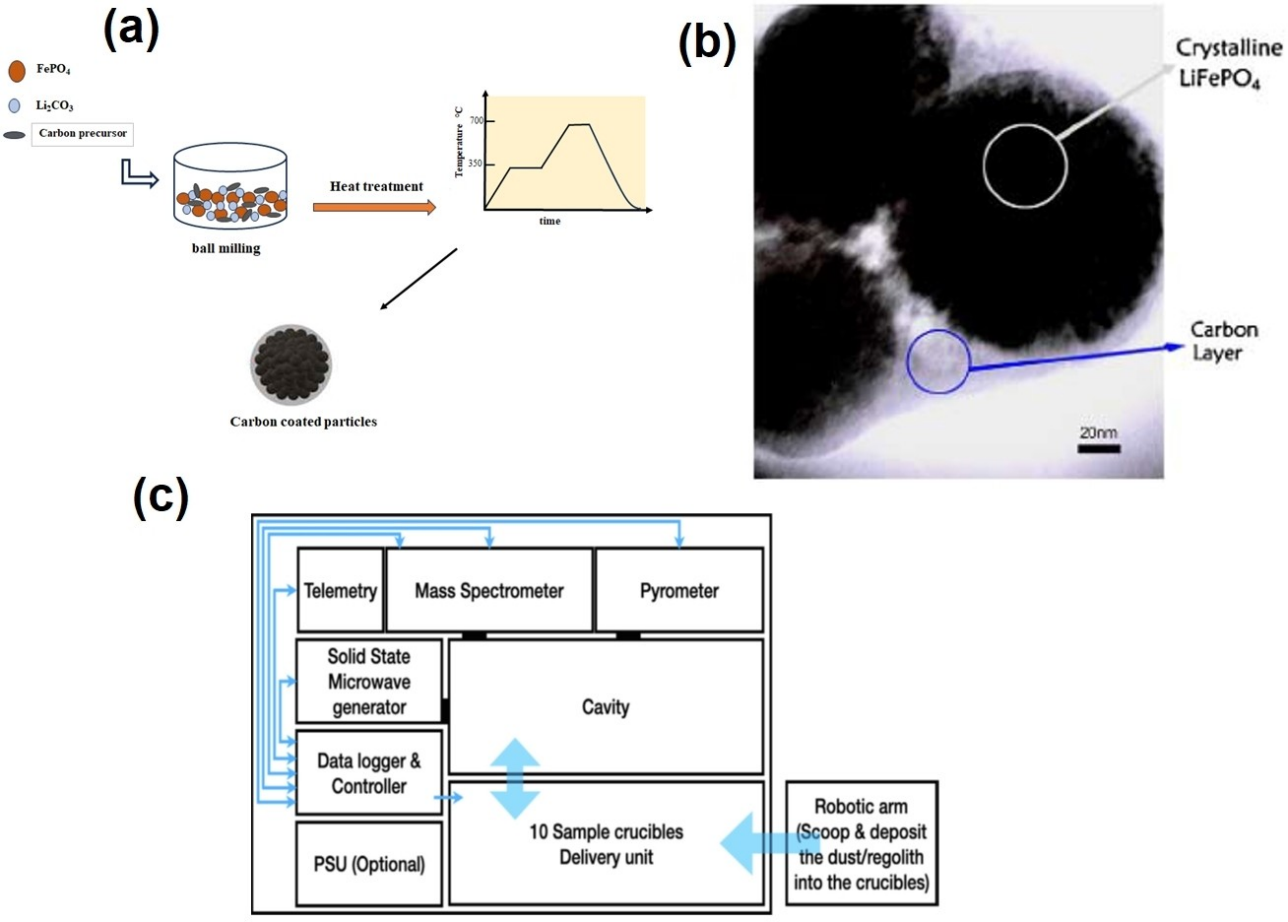
(a) Schematic illustration of solid‐state synthesis for LFP, (b) TEM image of LiFePO_4_‐PAS prepared at 700 °C for 4 h. Copyright 2008 Elsevier. Reproduced with permission from reference^[30]^ and (c) a planar diagram illustrating the process of microwave heating. Copyright 2021 Elsevier. Reproduced with permission from reference.^[27]^

Solid‐state synthesis is a traditional approach for producing ceramics, involving multiple steps of thorough grinding and heating the precise mixture of starting materials. This mixture typically comprises iron salt, such as Fe(II)‐acetate or Fe(II)‐oxalate, a lithium salt like lithium carbonate or lithium hydroxide, and often includes ammonium phosphate as a source of phosphorus.[^13^, ^14^, ^15^, ^16^, ^17^] The reaction mixture is initially heated at 300–400 °C in an inert atmosphere to decompose the ligands. After regrinding, the powder is reheated at 400–800 °C for 10–24 h. Before the second grinding step, a carbon‐containing compound, such as carboxylic acid, can be added to the precursor. This compound can serve as a carbon source in the LiFePO_4_/C composite. The schematic presentation is given in Figure 2a.

The purity of the material relies on growth parameters such as calcination temperature and exposure time. During calcination, an inert nitrogen or argon or slightly reductive atmosphere (argon or nitrogen with added hydrogen) is necessary due to the iron oxidation state (+2). However, residual Fe^3+^ phase presence is possible and frequently observed. Calcination above 800 °C results in the formation of both trivalent Fe_2_O_3_ and Li_3_Fe_2_(PO_4_)_3_. Trivalent Fe may form due to oxygen in the inert gas flow or residual air trapped in particle pores. Another drawback of this method is uncontrollable particle size growth and agglomeration, limiting the application potential of larger particles due to their small surface area. Despite being an effective and easily industrialized method, the solid‐state synthesis requires repeated reheating and regrinding to enhance final product homogeneity, rendering the processing time and energy‐consuming.[^13^, ^17^, ^18^] Additionally, researchers have modified traditional solid‐state reactions, achieving LiFePO_4_ preparation without using an inert gas flow. Researchers synthesized the LiFePO_4_ by heating Fe_2_O_3_, NH_4_H_2_PO_4_, and LiOH in deionized water for three hours at 700 °C.^[19]^

#### 2.1.1 Carbothermal Reduction

Low electronic conductivity is the main problem, which makes it challenging to use LiFePO_4_ as a battery cathode chemical in high‐power applications such as HEVs. Several studies have shown that the synthesis of LiFePO_4_ with a coating of an electronic conductor, such as a carbon layer, is an effective way to increase the electrochemical properties of LiFePO_4_. The TEM image in Figure 2b shows how the carbon layer covers the LiFePO_4_ particles. Several studies have been done to determine the best process for synthesizing LiFePO_4_‐C. Mi *et al*. have presented a one‐step process for synthesizing carbon‐coated LiFePO_4_ cathodes.^[20]^ The starting mixture consists of stoichiometric amounts of inexpensive FePO_4_ ⋅4H_2_O and LiOH⋅ H_2_O. The starting mixture was ball‐milled for 2 h in a planetary mill with a nylon vessel and then mixed with polypropylene as a carbon source and reductive agent. The mixture was annealed at 650 °C for 10 h in a nitrogen atmosphere to synthesize carbon‐coated LiFePO_4_ powder.^[20]^

High‐performance LiFePO_4_ cathode materials were produced through an advanced solid‐state method utilizing starch and PEG6000 as a complex carbon source. The wet‐mixing process in de‐ionized water at 80 °C enhanced precursor stabilization during drying and addressed challenges associated with mixing in a water medium. The starch gelatinization at 80 °C resulted in a gel emulsion precursor, which, after concentrated sedimentation during the drying process at 120 °C, transformed into a compact dry gel. Finally, the obtained dry gel was sintered at 700 °C for 12 h to yield the LiFePO_4_/C composite.^[21]^ A study presents an efficient supercritical CO_2_ (SCCO_2_)‐assisted ex‐situ carbon‐coating method for LFP. SCCO_2_ enhances carbon precursor penetration, yielding high‐quality carbon‐coated LFP. The resulting layer has a higher graphitic carbon content and fewer oxygen‐derived groups, benefiting electron transport.^[22]^ Additionally, an effective approach to addressing the slow kinetics of LiFePO_4_ involves integrating it with large contact area materials, such as graphene and carbon nanotubes, to create a large contact area. This approach is widely used to develop high‐rate hybrid electrodes, aiming to minimize the carbon content in the resulting materials.[^23^, ^24^]

#### 2.1.2 Microwave Heating

Microwave processing achieves rapid, uniform heating in ceramics at lower temperatures than traditional methods. It has been successfully applied in novel sintering for various materials.^[25]^ Microwave heating differs from conventional methods, which heat materials externally internally; instead, microwave heating occurs at the molecular level, ensuring a uniform temperature distribution throughout the material.^[26]^ Furthermore, this approach demonstrates high repeatability and eliminates the requirement for reducing gas. Cost‐effective carbon, characterized by fast heat production, is selected as the microwave absorbent to ensure effective heat generation. Carbon can also generate reduced gas, protect ferrous ions and eliminate iron ions. Figure 2c provides a schematic representation of microwave heating.^[27]^ Carbon is a popular microwave absorber due to its affordability and rapid heating properties, forming a reductive atmosphere to protect Fe(II) and prevent impurities during LiFePO_4_ powder production. While carbon is the primary microwave absorber, other materials like Fe, glucose, and yeast cells can improve heat generation efficiency and influence particle size, Li diffusion coefficient, and capacity loss, resulting in larger particle sizes and potential impurities.[^28^, ^29^]

### 2.2 Wet Chemical Processing

Wet chemical preparation methods, such as hydrothermal/solvothermal, sol‐gel, or co‐precipitation techniques, possess a distinct advantage over solid‐state reactions in achieving enhanced homogeneity and thorough mixing of the initial compounds at the molecular level.

#### 2.2.1 Hydrothermal (Solvothermal) Synthesis

**Figure 3:**
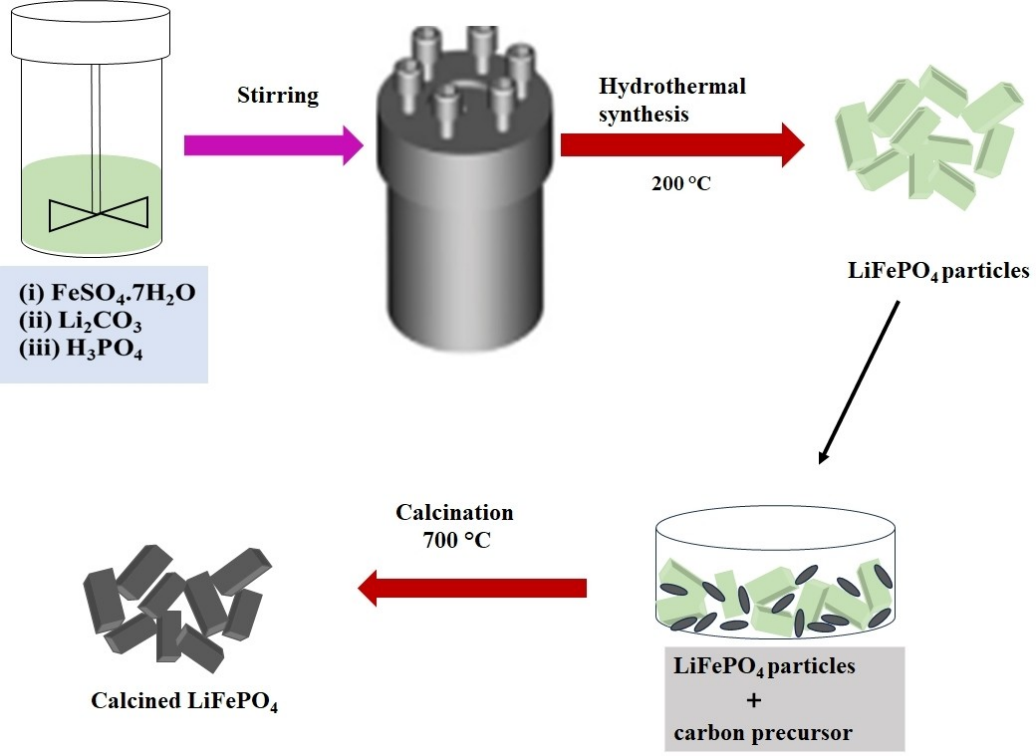
Schematic illustration of hydrothermal synthesis of LFP from iron sulfate.

A fast, simple, cost‐effective, energy‐efficient, and easily scalable method for generating small particles is hydrothermal or solvothermal synthesis. The synthesis of lithium iron phosphate via hydrothermal methods was initially demonstrated by mixing FeSO_4_, H_3_PO_4_, and LiOH in a molar ratio of 1 : 1 : 3. To prevent the formation of Fe(OH)_2_, which readily oxidizes to FeO(OH), the FeSO_4_ and H_3_PO_4_ solution were initially combined. Then, LiOH solution was added to the mixture, followed by hydrothermal processing at 120 °C for up to 5 h.^[31]^ Nevertheless, the resulting lithium‐iron phosphate phase did not exhibit a high capacity due to the disorder in the arrangement of lithium and iron, with iron occupying the lithium sites.^[32]^ The results showed that iron prevents lithium from moving through the one‐dimensional channels in the structure. Hence, it is essential to ensure that iron and lithium are correctly arranged. The problem was fixed when carbonaceous materials were heated to 700 °C for the hydrothermal material.^[33]^ During the study in 2003, the researchers concluded that “hydrothermal synthesis is not a feasible approach for lithium iron phosphate”,^[33]^ however, through subsequent modifications to the synthesis conditions, Chen et al. identified optimal hydrothermal parameters for producing electrochemically active LiFePO_4_.[^34^, ^35^] It was determined that a synthesis temperature exceeding 175 °C is necessary to reduce iron disorder and achieve proper lattice parameters and volume. Additionally, reductants like ascorbic acid or sugar prevent the formation of surface ferric films.[^36^, ^37^] Chen et al. noted hydrothermal synthesis produces well‐crystallized material with micron‐sized particles, typically a few hundred nanometers thick, where the short dimension aligns with the diffusion direction Figure 3.^[37]^

**Figure 4:**
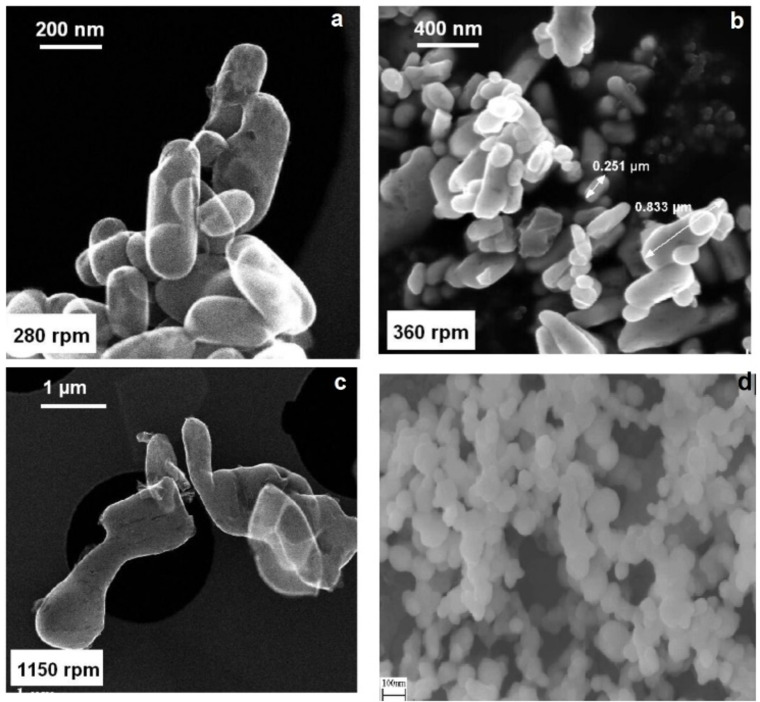
SEM image (a–c) of LFP synthesized using continuous stirring hydrothermal method at 280, 360, and 1150 rpm speed. Copyright 2014 Elsevier. Reproduced with permission from reference^[45]^ and (d) LFP synthesized in continuous hydrothermal synthesis at 573 K. Copyright 2007 Elsevier. Reproduced with permission from.^[40]^

In 2005, Lee et al. found that LiFePO_4_ formation in supercritical water occurred within a narrow pH range under neutral or slightly basic conditions, with pH minimally affecting particle size or shape.^[38]^ Moreover, they observed that smaller and more uniform particles were obtained in supercritical water synthesis compared to subcritical water.^[39]^ In addition to batch hydrothermal synthesis, the continuous hydrothermal technique was utilized to synthesize pure lithium iron phosphate nanoparticles; see Figure 4 d. Also, the water flow rate significantly influences particle morphology, and continuous hydrothermal synthesis yields smaller and more uniform particles than batch synthesis.^[40]^ Water temperature significantly affects the hydrothermal synthesis process for LiFePO_4_ powders, influencing their reaction rate, ionization degree, particle size, and crystalline structure. The structure and electrochemical properties are notably influenced by water flow rate and precursor concentrations.^[41]^ In addition, various carbon sources, such as sugar, ascorbic acid, carbon, multi‐walled carbon nanotubes (MWCNTs), and the organic surfactant acetyl trimethyl ammonium bromide (CTAB), serve as reducing agents during calcination to prevent the oxidation of Fe(II).^[42]^ Hydrothermal synthesis conducted with an organic surfactant compound like CTAB facilitated the production of lithium iron phosphate powders with high surface areas and enhanced electrochemical performance.^[43]^ CTAB pyrolysis produced a carbon layer on the particle surface, generating a reducing environment that prevented Fe^2+^ oxidation.

Additionally, hydrothermal synthesis is suitable for preparing metal‐doped lithium iron phosphates.^[44]^ It was revealed that the rotation speed in the reactor significantly affects the formation of secondary particles, as evidenced by Figure 4(a–c), SEM images of samples prepared at various speeds. At 280 rpm, primary particles take on an elongated spheroidal shape. This non‐spherical morphology, consistent at 360 rpm, is attributed to the olivine structure's geometry. Conversely, the sample prepared at 1150 rpm exhibits a distinct morphology. The findings suggest that stirring at 280 rpm is optimal for producing powder that enhances electrochemical properties.^[45]^

**Figure 5:**
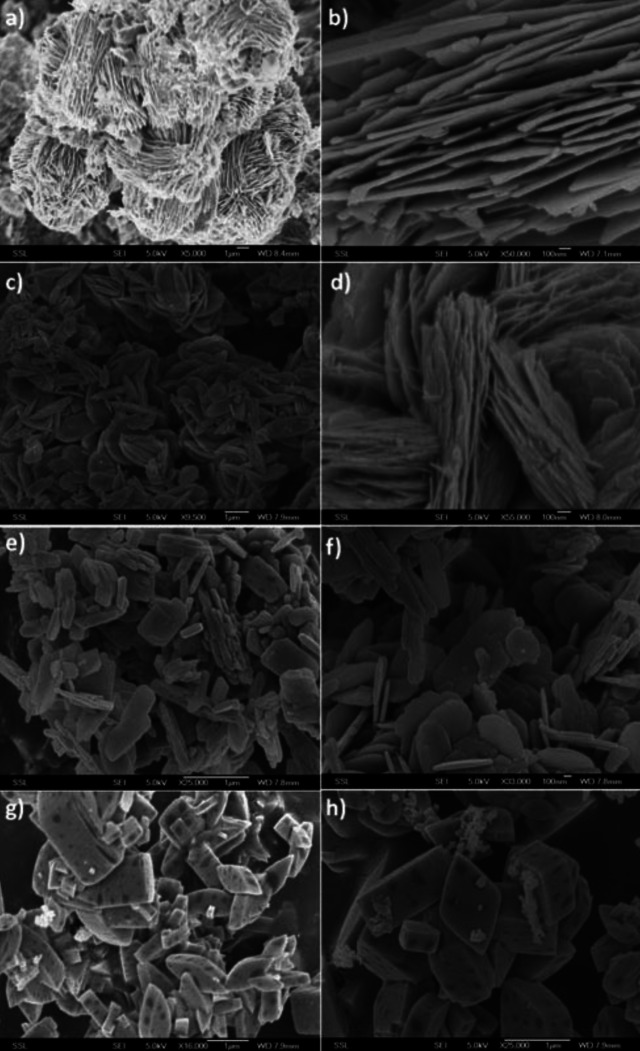
Morphologies of LFP using hydrothermal synthesis prepared from (a,b) Fe(oxalate), (c,d) Fe(acac)_3_, (e,f) Fe‐PMIDA, and (g,h) Fe(gluconate)_2_. Copyright 2010 Royal Society of Chemistry. Reproduced with permission from reference.^[46]^

LFP obtained from various iron precursors highlights the substantial impact of precursor selection on the synthesized material's morphology (Figure 5). Notably, iron oxalate resulted in hierarchical self‐assembled nanoplates, Fe(acac)_3_ led to the formation of thick plates with aggregation and a spindle‐like morphology, and Fe(gluconate)_2_ produced microcrystals shaped like diamonds.^[46]^

#### 2.2.2 Sol‐Gel Method

Sol‐gel processing of inorganic ceramic and glass materials, dating back to the mid‐1800s, is primarily motivated by its potential for higher purity and homogeneity alongside lower processing temperatures than traditional ceramic powder methods.^[47]^ Sol‐gel processing is driven by its potential for higher purity, homogeneity, and lower processing temperatures compared to traditional solid‐state methods. Precursors like lithium acetate, iron (II) acetate, lithium phosphate, iron (III) citrate, lithium oxalate, and iron (II) oxalate are commonly used in this method. Sol‐gel processing enables precise manipulation of material structure at the nanometer level, starting from the early processing stages. Sols consist of colloidal particles dispersed in a liquid, whereas colloids are solid particles ranging from one to a hundred nanometers in diameter. Gels are interconnected, rigid networks with submicrometer‐sized pores and polymeric chains. Gels encompass various combinations of substances, classified into four categories: well‐ordered lamellar structures, covalent polymeric networks, polymer networks formed through physical aggregation, and particular disordered structures. The precursors are dissolved in appropriate solvents, such as N,N‐dimethylformamide,,^[48]^ water with ascorbic acid, or citric acid,,[^49^, ^50^, ^51^] as chelating agent, ethylene glycol, and ethanol.^[52]^ Examples of combinations of lithium and iron sources include Lithium acetate and iron (II) acetate,[^48^, ^49^] lithium phosphate and iron citrate with phosphoric acid,^[50]^ lithium oxalate and iron (II) oxalate, lithium carbonate and iron (II) oxalate.^[51]^ The sols are converted into gels using standard procedures, followed by heating at temperatures between 500–700 °C in either an inert gas like argon or nitrogen or in a slightly reductive atmosphere containing 5–10 % hydrogen in argon/nitrogen.[^48^, ^52^] These temperatures are lower than those required by the solid‐state method, and typically, a one‐step heat treatment is adequate to produce phase pure LiFePO_4_. Besides improving precursor mixing for lower temperature synthesis compared to solid‐state methods, the sol‐gel method offers the advantage of coating evolving LiFePO_4_ particles with carbon from solvents or precursor ligands.^[51]^ Composites containing carbon and iron phosphides can also be produced using an aqueous sol‐gel method with ethylene glycol as a carbon source and a N_2_+5 vol% H_2_ calcination atmosphere. Citrate in gel preparation facilitates hierarchical pore formation in the meso and macro ranges.^[50]^ During high‐temperature treatment under inert or reducing atmospheres, decomposing organics coat the LiFePO_4_ crystals with an amorphous carbon surface layer. However, the presence of carbon coating reduces the density of LiFePO_4_, thereby decreasing the reversible capacity of the electrode with an increasing amount of carbon. The heating rate during calcination significantly influences the structure of LiFePO_4_ powders. A slow heating rate tends to produce a rougher and less porous structure. Conversely, a high heating rate leads to a more porous structure, impacting the electrochemical properties of the powders.^[42]^ In general, several precursors and solvents are used in Sol‐gel synthesis to produce LiFePO_4_ powders; the solvent used significantly impacts the powder's structure. Although organic solvents can also be employed, water is the most often utilized solvent.^[53]^

#### 2.2.3 Co‐Precipitation

Like sol‐gel, the co‐precipitation method improves purity, crystallization, and particle sizes at lower temps. Homogeneous lithium iron phosphate is synthesized through aqueous co‐precipitation of Fe^2+^ precursor and subsequent heat treatment in nitrogen.^[54]^ Researchers have noticed that raising the temperature of a solution containing Li^+^, Fe^2+^, and P^5+^ ions above 105 °C while adjusting the pH to between 6 and 10 promotes the formation of LiFePO_4_ rather than a mixture of Li_3_PO_4_ and Fe_3_(PO_4_)_2_. To elevate the solution temperature above that of pure water, water‐miscible boiling point elevation additives such as ethylene glycol, diethylene glycol, or N‐methyl formamide are introduced. Once the solution reaches the solvent's boiling point, LiFePO_4_ begins to precipitate. Subsequently, the obtained precipitate is calcined at 500 °C in a slightly reducing atmosphere.^[55]^ The precipitation step in LiFePO_4_ synthesis forms the crystalline phase, reducing the required thermal treatment time and temperature compared to ceramic synthesis.

Additionally, LiFePO_4_ can be synthesized via aqueous precipitation of FePO_4_⋅H_2_O followed by carbothermal reduction of a mixture containing iron (III) phosphate precipitate and lithium carbonate with carbon as the reducing agent.^[56]^ Nanocrystalline LiFePO_4_ with enhanced electrochemical performance was synthesized through a two‐stage process involving lithiation of FePO_4_⋅xH_2_O with oxalic acid followed by calcination at 500 °C.^[57]^ LiFePO_4_/C composite was successfully produced via sonochemical precipitation and calcination, where Li_3_PO_4_ and FeSO_4_ in a PVA aqueous solution underwent sonochemical treatment, followed by calcination at 600 °C.^[58]^ As a result, both calcination and synthesis times were reduced, yielding a powder of LiFePO4 particles coated with carbon that exhibited significant discharge capacity with excellent retention.^[58]^ LiFePO_4_ nanoplates (<50 nm thickness) synthesized via coprecipitation in ethylene glycol at 180 °C show rapid crystallization within 20 min. Feeding sequence influences crystal growth, allowing tuning of orientation and particle size.^[59]^ LiFePO_4_ polycrystalline material synthesized via an eco‐friendly co‐precipitation method shows amorphous structure and stable performance up to 800 °C. The material exhibits electronic suitability with a band gap of 4.1 eV and evolving Li^+^ ions conductivity for potential electronic applications.^[60]^ Moreover, coprecipitation can be combined with other techniques to produce LiFePO_4_ powders with controlled structures and functionalities.^[61]^ Improving the structure and functionality of LiFePO_4_ powders is achieved by incorporating carbon or metal dopants, effectively addressing challenges such as poor electronic conductivity and low Li^+^ diffusion rates. This synergistic effect results in improved electrochemical reactivity and enhanced overall performance in Li‐ion batteries.^[62]^

#### 2.2.4 Ultrasonic Spray Pyrolysis

Ultrasonic spray pyrolysis generates droplets efficiently, offering simplicity, cost‐effectiveness, and extensive surface coverage. Despite challenges in small droplet removal, it delivers stable coatings, versatility in precursor solutions, and overall cost‐effective applications. Moreover, the process is highly effective in swiftly producing fine, clean, and well‐crystallized ceramic particles with controlled size distribution, ensuring high purity, easy synthesis process control, and precise particle size dispersion. Spray pyrolysis holds significance as a technique for producing ultrafine powders, providing clear advantages in both process control and material quality.^[63]^ The previously described characteristics make the spray pyrolysis process a good option in academics to produce LiFePO_4_ powders to improve electrochemical performance.[^64^, ^65^, ^66^, ^67^] The synthesis process involves atomizing a precursor solution using an ultrasonic nebulizer, with the sprayed droplets transported to the reactor by gas. The reactor temperature varies from 400–600 °C, and the resulting powders undergo additional sintering at 600–800 °C temperatures. Spray pyrolysis with carbon sources can produce LiFePO_4_/C powders with lower particle sizes, increasing specific surface area. Carbon‐coated three‐dimensional porous LiFePO_4_ microspheres are made in a fast processing time (about 10 min) with improved electrochemical characteristics, consistent shape, and 60 μm size by the use of a supercritical and spray‐dry combination approach.^[68]^ Kashi et al. employed table sugar, citric acid, and sucrose as carbon precursors during ultrasonic spray pyrolysis and calcination to synthesize LFP−C samples. The addition of sucrose effectively prevents agglomeration during calcination, leading to a higher carbon content. Electrochemical experiments for Li‐ion storage show inferior performance in carbon‐free and uncalcined samples. However, samples containing 2.5 and 5.0 g of sucrose per 100 ml LFP precursor solution demonstrate excellent results.^[66]^ Metal dopants also ensure high tap density and short production time for commercially viable electrode materials. Synthesized LiFeTMPO_4_ nanoparticles by spray pyrolysis, especially LiMn_1/3_Fe_2/3_PO_4_, demonstrate improved electrochemical performance and higher practical energy density for cost‐effective and high‐performance battery materials.^[69]^

#### 2.2.5 Other Methods for Synthesis

In addition to the mentioned techniques, the quest for innovative and alternative methodologies continues. Freeze‐drying represents one such approach. The LiFePO_4_/C composite was synthesized using LiH_2_PO_4_, FeC_6_H_5_O_7_, and nitrogen‐doped carbon nanotubes. After mixing the raw materials and N‐CNTs in water, the suspension was frozen with liquid nitrogen and dried in a vacuum freezer. The solidified precursor was heated in a tubular furnace under a reducing atmosphere to form the composite.

Similarly, pristine LiFePO_4_ and LiFePO_4_/C composites were prepared without N‐CNTs or with regular CNTs.^[70]^ While solid‐state reaction is commonly used for commercial LiFePO_4_ production, alternative, more cost‐effective synthesis methods are being explored. However, both LiFePO_4_ and FePO_4_ phases exhibit poor electronic conductivity due to singular Fe cation oxidation states (2^+^ or 3^+^), and limited lithium‐ion movement in one‐dimensional tunnels hampers achieving high energy density. Defects and impurities further hinder performance, particularly at high currents.

By offering insights into the optimal approach based on the synthesis process and synthesis product, this research is anticipated to positively affect the LiFePO_4_ manufacturing sector. Table 1 presents a comparative analysis of many techniques.

**Table 1:** A comparative analysis of the LiFePO_4_ synthesis methods.

Method	Materials & concentration	Experiment condition	Advantages	Results	Discharge Capacity	Ref
**Microwave heating**	‐ Li_2_CO_3_ (99 %) ‐ NH_4_H_2_PO_4_ (99 %) ‐ Fe(CH_3_COO)_2_ (95 %)	5–20 min in 2.45 GHz, with a power level of 500 W	*Short reaction time *Microwave heating is a promising method.	*High electrochemical capacity and good cycle ability * The discharge capacity ~125 mAh/g	125 mAh/g at 10 mA/g and 60 °C	^[25]^
**Solid‐state**	‐ α‐Fe_2_O_3_, ‐ LiOH ⋅ H_2_O ‐ NH_4_2HPO_4_ ‐ 5 wt % glucose and 40 wt % oxalic acid	‐ 700 °C for 3 h without nitrogen gas flow	* Smaller particles size for LiFePO_4_ prepared from NH_4_H_2_PO_4_ to that prepared from (NH_4_)_2_HPO_4_	* A better electrochemical performance in the sample prepared from NH_4_H_2_PO_4_	138 mAh/g at 0.2 C and 25 °C	^[19]^
**Carbothermal reduction**	‐ FePO_4_⋅4H_2_O ‐ LiOH⋅H_2_O ‐ Polypropylene (PP)	‐ FePO_4_ and LiOH ball‐milled for 2 h ‐ 36.8 g of PP per mole FePO_4_ at 650 °C for 10 h	* one‐step reaction * Suitable for mass production of LiFePO_4_/C	* Discharge capacity of 160 mAh/g at 0.1 C in 30 °C.	160 mAh/g at 0.1 C and 30 °C	^[20]^
**Solid‐state reaction**	‐ LiOH⋅H_2_O ‐ Fe_2_O_3_ ‐ H_3_PO_4_	calcined at 700 °C for 2 h and sintered at 900 °C for 6 h.	* Produce particle ranges from micrometers to nanometers * Produce single or multi component particles * Suitable for industrial application	* Active ingredient LiFePO_4_ synthesized without carbon coating * The final phase of LiFePO_4_ has a space group Pnma, indicating an olivine structure.	154 mAh/g at 0.2 C and 25 °C	^[71]^
**Carbothermal reduction**	‐ FePO_4_ ‐ Li_2_CO_3_ ‐ Sucrose	‐ Calcined at 350 °C for 5 h under N_2_ atmosphere. ‐ Took out the sample and ground it. ‐ Calcined again at 750 °C for 10 h under N_2_ flow.	* Controlled morphology for LiFePO_4_/C * Understanding how small particles fill voids formed by large particles, improving tap density * Improved tap density and specific capacity	* Spherical particle morphology improves tap density and high‐rate performance * Tap density: 1.3649 g cm^−3^ and specific capacity of 94.85 mA h/g at 5 C.	164.8 mAh/g at 0.1 C and 25 °C	^[72]^
**Solvothermal**	‐ 15 mmol LiOH.H_2_O ‐ 5.5 mmol H_3_PO_4_ ‐5 mmol FeSO_4_.7H_2_O ‐ Sucrose for carbonization	‐ 10 h in 180 °C for heat treatment ‐ 3 h in 550 °C for carbonization	*Using low temperature *No other impurities	*Uniform synthesis of nanorod LiFePO4 via solvothermal processes *Good rate and cycle performance at room temperature	167 mAh/g at 0.2 C and 25 °C	^[73]^
**Hydrothermal**	‐ FeSO_4_⋅7H_2_O ‐ H_3_PO_4_ (85 %) ‐ LiOH (98 %) ‐ ethylene glycol with a molar ratio of 3 : 1 : 1 : 1/3.	‐ Sonication process in 30 min ‐ Heating in autoclave at 180 °C for 24 h. filter, dry. ‐ Heating at 600 °C for 6 h.	* Reducing energy consumption * Inexpensive, reducing overall production costs * Uniform particle size distribution, enhancing product quality	* Sonication improved cathode performance. * Smaller particles in the raw material mixture led to more uniform growth of LFP crystals.	125 mAh/g at 0.1 C and 25 °C	^[74]^
**Sol‐gel**	Stoichiometric amounts of: ‐ LiCH_3_COO ‐ Fe(CH_3_COO)_2_ ‐ H_3_PO_4_ ‐ C_6_H_10_O_4_ ‐ Adipic acid	‐ Dissolved in ethanol and evaporate at 90 °C for 4 h. dry. ‐ Calcined at 400 °C for 1.5 h. ‐ Calcined again at 650–700 °C for 2.5 h.	* Ideal for large‐scale production of LiFePO4 particles with high rate retention and capability. * Low calcination temperature, high yield, and excellent cycle characterization	* LFP particles obtained range between 50 and 100 nm. * LFP cells exhibit an initial discharge capacity of >150 mAh/g at 50 °C. * No capacity fading at ambient temperature for up to 100 cycles.	159 mAh/g at 0.2 C and 25 °C	^[75]^
**Sol‐gel**	‐ C6H8O7 (1.455 g) ‐ FeC6H5O7 (1.106 g) ‐ Li3PO4 (161.4 mg), ‐ NH_4_H_2_PO_4_ (345 mg) ‐ Ttissue paper MWCNT (100 mg)	‐ Sonication for 2 h in 10 ml H_2_O. ‐ Add tissue paper and stirred at 60 °C until gel. ‐ Gel dried and calcinates at 600, 700, 800 °C.	* Use of tissue paper as a template * Sol‐gel process for microstructure synthesis * Tape‐casting process for LiFePO4‐MWCNT sheets	* Single‐phase of orthorhombic olivine structured LFP. * Authenticated formation of 1D LiFePO4 microstructures and particle clusters. * Enhanced LiFePO4‐MWCNT electrochemistry.	115 mAh/g at 0.1 C	^[76]^
**Co‐precipitation**	‐ Fe(NO_3_)_3_ ‐ LiNO_3_ ‐ (NH_4_)_2_HPO_4_ ‐ ascorbic acid ‐ ammonia ‐ Sugar (20 wt % of LFP)	‐ Co‐precipitated powder dispersed in sugar solution, then dry and heat. ‐ Calcined at 350 °C for 10 h. ‐ Sintered at 600 °C for 16 h in nitrogen flow.	* Inexpensive precursors used. * Only water vapor produced, making the process environmentally friendly * LiFePO4 powder well distributed, avoiding agglomeration	* Homogeneous synthesis of pure olivine LiFePO4. * Excellent stability * Conductivity of LFP/C composite increased due to carbon scaffold.	154 mAh/g at 0.2 C and 25 °C	^[77]^
**Co‐precipitation**	‐ Fe(NO_3_)_3_ (1 M) ‐ H_2_O_2_ (1 M) ‐ (NH_4_)_2_HPO_4_ (1 M) ‐ Li_2_CO_3_ ‐ Citric acid	‐ Hydrothermal mixed continuous at 3000 rpm. ‐ Precipitates from the outlet to batch reactor at 300 rpm for 3 h. ‐ Anhydrous FePO_4_, Li_2_CO_3_, and citric acid mixed and calcined at 650 °C for 10 h.	* Innovative High Mixing Continuous Rotating Reactor (HMCRR) Technology. * LiFePO_4_/C with mesoporous structure, larger specific surface area, and more uniform morphology composed of nano primary particles compared to traditional methods.	* Di‐hydrous FePO_4_ with micro‐spherical particles composed of uniform nanoplates with excellent crystallinity. * High‐rate capacity with discharge capacity at 10 C reaching around 125.4 mAh g^−1^. * Enhanced properties of FePO_4_ inherited by LiFePO_4_/C prepared by HMCRR, showing improved cycling stability and outstanding high‐rate capacity.	162 mAh/g at 0.1 C and 25 °C	^[78]^
**Ultrasonic Spray Drying Method**	‐ LiH_2_PO_4_ (0.036 mol) ‐ FeCl_2_ (0.036 mol) ‐ LiOH (0.00108 mol) ‐ HCl (15 ml 37 %) ‐ Sucrose (0.85188 g)	‐ Dissolved in 50 mL deionized water and then using ultrasonic atomizer. ‐ Tube furnace at 200 °C–350 °C. ‐ Spray dried LFP powder for 1 h. ‐ Tube furnace at 650 °C for 8 h.	* Convenient home‐made spray drying equipment. * Spray drying temperature significantly impacted memory effect and specific capacity of electrode materials.	* Memory effect enhanced from 1.3 mV–2.9 mV as spray drying temperature increased. * Defect of Li–Fe anti‐site blocked some [010] channels of LiFePO4 structure, causing retardation of Li‐ion migration. * Specific capacity reduced from 161 mAh/g–151 mAh/g as spray temperature increased.	161 mAh/g at 0.1 C	^[79]^
**Freeze‐drying**	Stoichiometric amounts (10^−3^ mol) of LFP: ‐ C_6_H_8_O_7_.H_2_O ‐ FeC_4_H_6_O_4_ ‐ LiOH.H_2_O ‐ NH_4_.H_2_PO_4_ (1 : 1 : 1 : 1)	‐ Dissolved in 25 mL water and frozen drop‐by‐drop under liquid N_2_. ‐ Freeze‐dried for 48 h in Telstar Lab Freeze‐Dryer. ‐ Calcined twice: 350 °C and 600°.	* Effective synthesis process using freeze‐drying method. * Homogeneous carbon covered LiFePO_4_ sample.	* Specific capacity value of 164 mAh g^−1^ obtained at C/40 rate. * The capacity retention study showed very promising results, with more than 97 % retention after 50 cycles. * Good reversibility of the material demonstrated by cyclic voltammetry.	146 mAh/g at 0.1 C and 25 °C	^[80]^
**Freeze‐drying**	Stoichiometric amounts (10^−2^ mol) of LFP: ‐ LiH2PO4 ‐ FeC6H5O7 ‐ N‐doped Carbon Nanotubes	‐ Dissolved in 20 mL water while stirring. ‐ Instant freezing of the suspension under liquid N_2_ ‐ drying at −55 °C in a vacuum freezer for 48 h. ‐ Heated in a tubular furnace at 650 °C under a reducing atmosphere (H_2_/Ar, 10/90) for 10 h.	* Three‐dimensional porous LiFePO4 modified with uniformly dispersed nitrogen‐doped carbon nanotubes. * Superior three‐dimensional conductive network enhancing electronic conductivity and accelerating lithium‐ion diffusion.	* Uniform dispersion of nitrogen‐doped carbon nanotubes inside the porous LiFePO_4_. * Excellent electrochemical performance in terms of charge/discharge tests, and electrochemical impedance spectroscopy.	159 mAh/g at 0.1 C and 25 °C	^[70]^

## 3 Recycling Processes

### 3.1 General

The recycling process for LiFePO_4_ batteries encompasses several essential steps, including battery collection, disassembly, crushing, component separation, metal recovery, and the environmentally friendly disposal of non‐metallic materials. By following these processes, recycling LiFePO_4_ batteries brings notable benefits like decreasing the need for raw materials, preserving energy, and reducing greenhouse gas emissions linked to battery production.

After collection, the batteries undergo crushing to create small fragments, followed by component separation using magnetic separation, sieving, and chemical processes. The recovered metals, like lithium, iron, copper, and aluminum, undergo further processing to obtain high‐purity materials suitable for reuse. Meanwhile, non‐metallic items such as plastics, papers, and electrolytes are disposed of properly to safeguard the environment.

**Figure 6:**
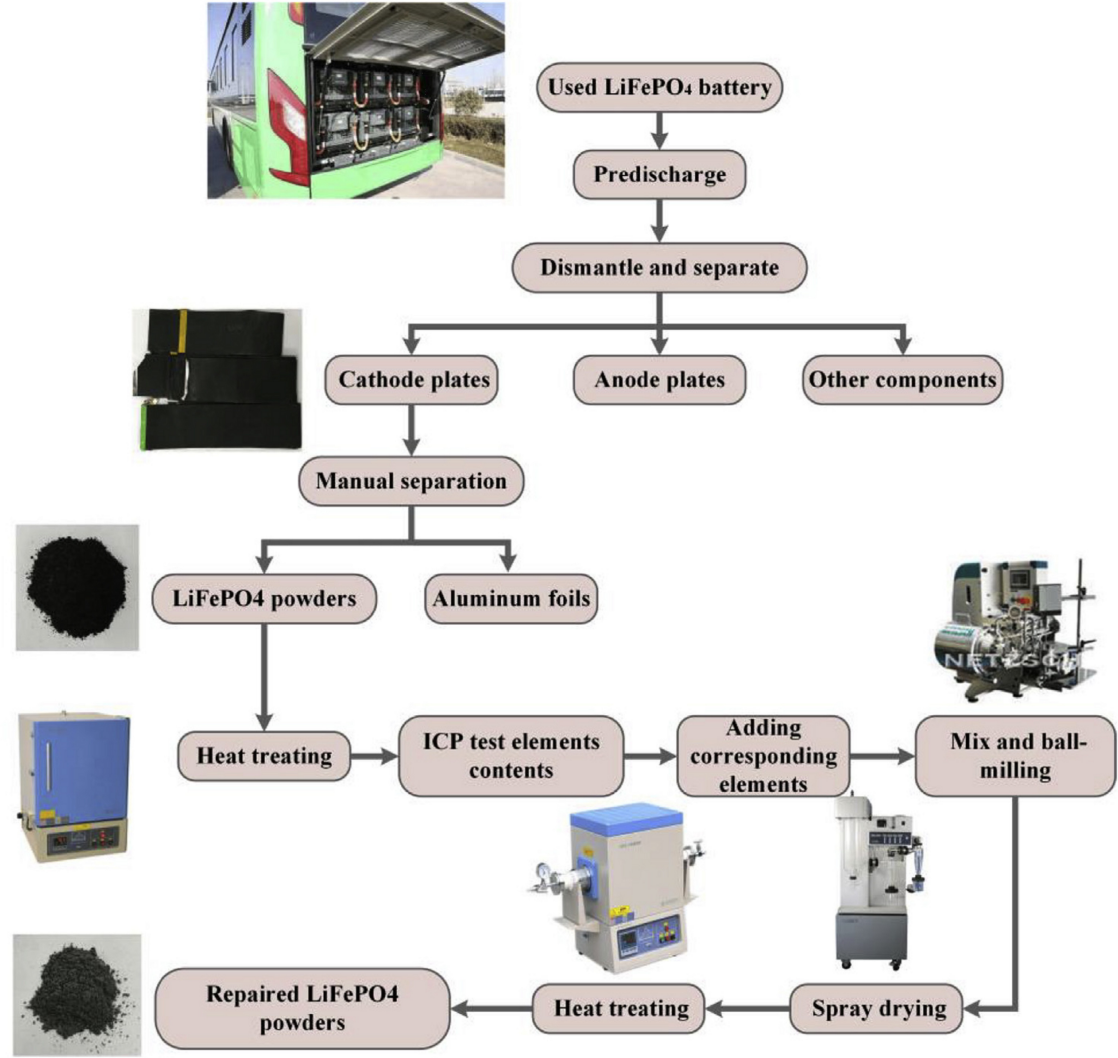
The recycling and repair processes for spent LFP. Copyright 2019 Elsevier. Reproduced with permission from reference.^[81]^

Figure 6 describes the recycling processes for used LiFePO_4_ batteries. After being retired from an electric vehicle, the used battery undergoes capacity detection and pre‐discharging to ensure safety. It is then broken down into separate anode plates, cathode plates, and other parts for treatment. The cathode plates are cleaned and dried to produce aluminum foils and LiFePO_4_ powders. These powders are further processed through ball‐milling and spray drying after being annealed to remove impurities and tested for element content.^[81]^ Following the initial pretreatment, active materials in spent LiFePO_4_ batteries are separated using chemical and physical methods like thermal treatment, alkaline leaching, or organic solvent. Thermal processes sort materials based on their melting points, while the chemical method involves dissolving aluminum foil in an alkali solution to obtain LiFePO_4_, collected through filtration. Recycling of LiFePO_4_ batteries involves three main approaches: recovering valuable metals, regenerating and utilizing LiFePO_4_, and preparing lithium ferrite.^[82]^

### 3.2 Recycling Strategies of Spent LiFePO_4_ Batteries

Pyrometallurgy and hydrometallurgy are commonly employed methods for recycling used batteries. Pyrometallurgy involves subjecting used lithium‐ion batteries (LIBs) to high temperatures, leading to their physical breakdown. By adjusting the molar ratio and subsequently recrystallizing and renewing the constituent parts, valuable metals and components within the spent LIBs can be recovered. On the other hand, the hydrometallurgical process focuses on the recovery of individual metals like lithium (Li), cobalt (Co), manganese (Mn), nickel (Ni), and iron (Fe) from old LIBs. This process begins by digesting the used batteries in an appropriate solvent, such as acid, alkaline, or natural organic acid, effectively dissolving the metallic elements in the batteries.[^83^, ^84^]

#### 3.2.1 Pyrometallurgy

This approach involves subjecting the material's metal oxides to high temperatures in a furnace to create an alloy using smelting and calcination. The pyrometallurgical technique effectively removes the separator, electrolyte, and binder from the battery through evaporation. The success of the process depends on various factors such as reaction time, temperature, and the type of purge gas used, which can impact the efficiency of metal recovery and may lead to the emission of hazardous gases. An optimal temperature is carefully selected to burn off organic compounds and polymers without causing undesirable phase changes. Despite some loss of components, the pyrometallurgy process is relatively safe and energy‐efficient, thanks to the occurrence of exothermic reactions. Furthermore, this method requires minimal pretreatment and is well‐suited for batteries that must be sorted appropriately.^[85]^ The widely used techniques for treating batteries include smelting, roasting/calcination, and pyrolysis. Additionally, there are ongoing studies on microwave‐assisted carbothermic reduction and salt‐assisted roasting. These methods are being explored for their effectiveness in battery recycling and waste management.^[86]^

Spent batteries are heated to temperatures as high as 1400 °C during the smelting process, which is higher than the melting point of metals and oxides. This leads to the creation of slag and molten alloys. For example, this method successfully recovered cathode materials like LCO, LFP, LMO, and NCM811. Co, Cu, Ni, Fe, and P were efficiently recovered as alloys, while Li and Mn were transformed into chloride and stored in the dust using CaCl_2_. The process achieved high Co, Cu, Ni, Fe, and P recovery within 80 min at 1450 °C.^[87]^ In a pyrometallurgical procedure created by Umicore, the spent LIBs were melted without prior preparation in a shaft furnace. An alloy made of Co, Cu, Ni, and Fe was created as a result of the method. However, lithium, manganese, and aluminum were lost in the slag without any attempt at recovery. Using chlorination roasting makes it possible to extract lithium from the slag.^[88]^

The roasting technique is performed at temperatures below the melting point, primarily aiming to reduce metals to a lower valence stage. An illustrative roasting technique is carbothermal reduction (CTR), in which reductant carbonaceous materials are mixed with the cathode material and subjected to elevated temperatures, typically ranging from 650–1000 °C.^[86]^ In salt‐assisted roasting, the separation of Li and LiFePO_4_ was done by sodium sulfate‐assisted roasting. After roasting, LFP transformed into iron compounds (Fe_2_O_3_ and FePO_4_) and water‐soluble LiNaSO_4_. Soaking the roasted material in water allowed solid‐liquid separation, yielding Li_3_PO_4_ and FePO_4_.^[89]^ The principal outcomes of both smelting and roasting methods encompass the production of metal alloys, slag, gases, and precursor materials that can be utilized in subsequent hydrometallurgical processes.

Pyrolysis is heating metal‐containing materials at high temperatures without oxygen, leading to decomposition and producing toxic gases. Vacuum pyrolysis, as an alternative approach, can prevent the release of harmful gases into the environment, but this technique consumes energy and necessitates expensive equipment.^[90]^ The Umicore process consumes 5000 MJ energy per ton of processed batteries.^[88]^

In the pyrometallurgical treatment of spent LIBs, the typical recovery rates for Li, Fe, and P can vary based on the specific process used. For instance, one study reported recovery rates of approximately 98.93 % for lithium using atmosphere‐assisted roasting.^[91]^ Zhang et al. studied the recycling of LFP batteries using a sodium salt‐assisted roasting method with two different sodium salts: Na_2_CO_3_ and NaOH. Na_2_CO_3_ was used to break down LFP, into iron and lithium salts. These were then separated by magnetic separation, achieving a 99.2 % lithium recovery rate. In the second approach, NaOH oxidized LFP into different compounds, resulting in a 92.7 % lithium recovery rate after magnetic separation.^[92]^ Li et al. used NaOH to oxidize Fe (II) in LFP at 150 °C, breaking down its structure and releasing Li and Fe. Magnetic separation yielded Fe_3_O_4_ and a lithium recovery of 96 %.^[93]^ A pyrometallurgical recycling process was tested using two reactor designs for recovering LFP with Al_2_O_3_ and MgO crucibles. In the Al_2_O_3_ crucible, lithium recovery was 68.4 %, and phosphorus recovery was 64.5 %. In the MgO crucible, phosphorus removal was 64 %, and lithium removal was 68 %.^[94]^

However, pyrometallurgical processes are not advised due to their high energy consumption, gas emissions, loss of lithium during recycling, and strict requirements for specialist treatment equipment.^[95]^ Hence, pyrometallurgy is not considered effective for recycling LFP batteries.

#### 3.2.2 Hydrometallurgy

Hydrometallurgy is essential for metal recycling and extraction, providing a more sustainable and efficient alternative to traditional pyrometallurgical methods.^[96]^ Hydrometallurgy is used to extract metals from ores or waste materials using liquid solutions. It involves dissolving metals in a liquid, separating and recovering the desired metals from the solution. Compared to pyrometallurgy, hydrometallurgy has lower energy consumption, reduces greenhouse gas emissions, and allows for the extraction of specific metals with greater efficiency.^[97]^ These techniques can be combined and adapted based on the application, metal recovery goals, and environmental considerations.^[98]^ Li^+^, Fe^2+^ or Fe^3+^ solutions are acquired by leaching materials with inorganic and organic acids or oxidation reagents. It is possible to use appropriate acids along with oxidizing agents like H_2_SO_3_, NH_2_OH, and H_2_O_2_ during the leaching process. Employing supporting agents during leaching can achieve more effective recovery of metal ions from spent LFP materials.^[85]^ There are two forms of leaching utilized for metal recovery: nonselective and selective recovery leaching. Li is recovered from the leaching solution in the selective recovery approach, and FePO_4_ is obtained as a leaching residue. In contrast, the nonselective strategy recovers the cathode into the leach solution, including Li, Fe, and PO_4_.^[99]^

##### 3.2.2.1 Inorganic Acid Leaching

The cost‐effectiveness and wide availability of H_2_SO_4_ make it stand out among mineral acids. It has also performed well in a variety of industrial applications. The selective one‐step acidic extraction of lithium (Li) from the Li‐Fe−P framework can be represented by the following reaction:

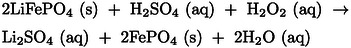




LiFePO_4_ and Al in Al‐bearing used LFP cathode powder recycled using H_2_SO_4_ leaching. The results demonstrated leaching efficiencies of 91.53 % for LFP and 15.98 % for Al under ideal conditions. Furthermore, lowering the leaching temperature effectively reduced Al dissolution during the acid leaching of the used LFP cathode material.^[100]^ During the leaching process, oxidizing agents like H_2_O_2_, H_2_SO_3_, and NH_2_OH can result in more efficient recovery of metal ions from spent LFP materials. For example, by employing stoichiometric H_2_SO_4_ and H_2_O_2_, Li can be selectively leached into the solution, while Fe and P are retained in the leaching residue as FePO_4_. Under the optimized conditions, the leaching rate for Li was 96.85 %. The recovered Li is precipitated as Li_3_PO_4_ using Na_3_PO_4_, and the FePO_4_ in the leaching residue is directly recovered through a burning process.^[101]^ Another study examines an optimized process for recovering LiFePO_4_ from spent lithium‐ion batteries using various surfactants (CTAB, SDS, and PEG) on recovered FePO_4_.2H_2_O. This approach achieves 98 % iron and 97 % lithium leaching when the mixture is dissolved in 2.5 M sulfuric acid.^[102]^

**Figure 7:**
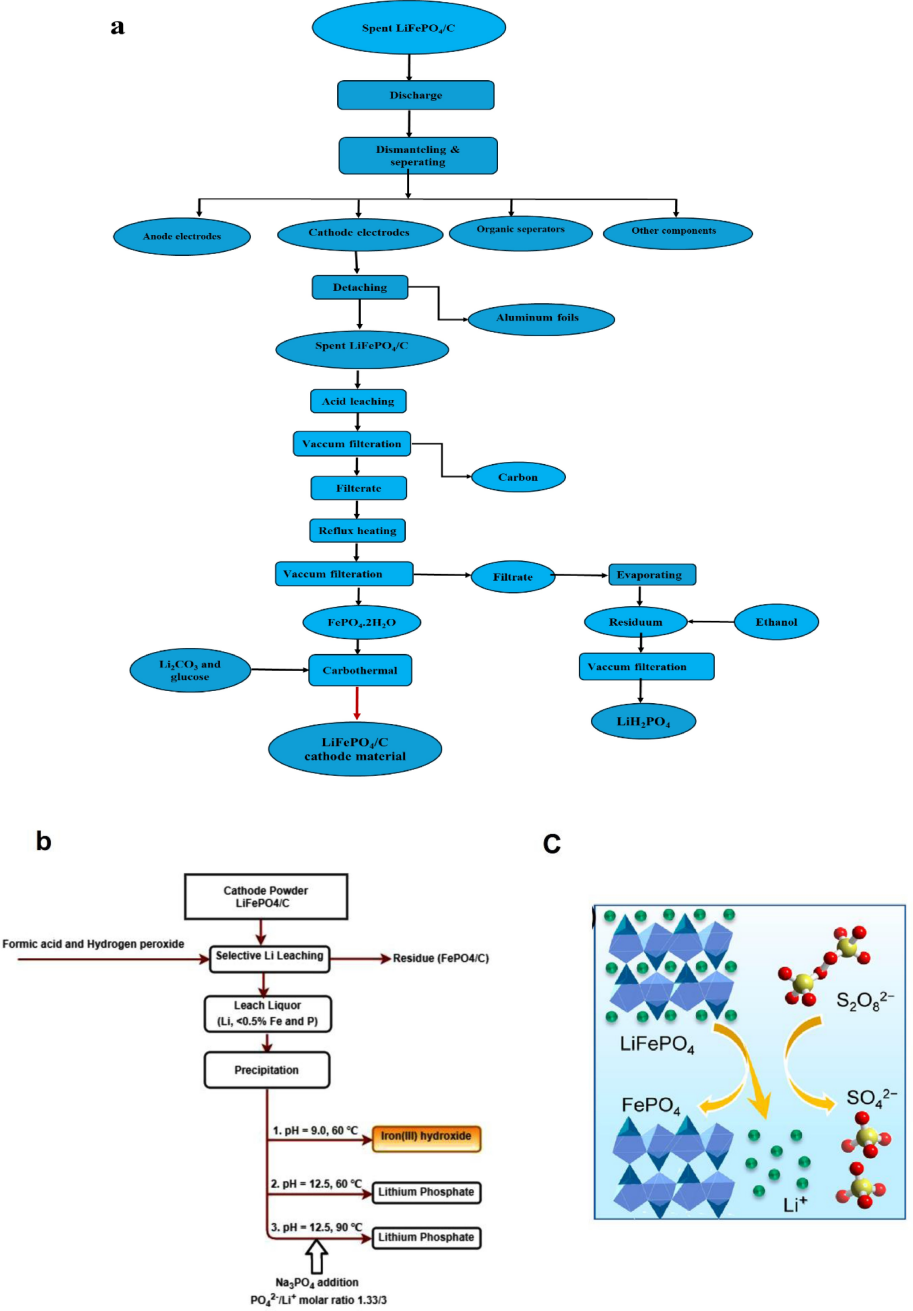
(a) Recycling process of spent LiFePO_4_ batteries using alkaline‐based leaching method. Copyright 2015 Elsevier. Reproduced with permission from reference,^[103]^ (b) diagram for recycling spent LiFePO_4_ batteries using the formic acid‐H_2_O_2_ leaching system. Copyright 2021 Elsevier. Reproduced with permission from reference,^[106]^ and (c) schematic illustration of the leaching mechanism that preserves the olivine crystal structure without alteration. Copyright 2019 American Chemical Society. Reproduced with permission from reference.^[110]^

Li, Fe, Al, and Cu can be recovered substantially from used LFP cathode material using the acid‐alkaline leaching method, resulting in a high retrieval rate. The approach involves three fundamental steps: thermal pretreatment, acid leaching, alkaline leaching, and precipitation. In the first step, the spent LFP cathode powder is heated at 600 °C for four hours, utilizing an H_2_SO_4_‐based acid to separate Al, Fe, and Cu. In the second step, the leaching residue undergoes alkaline‐based leaching to recover Li as LiH_2_PO_4_ through chemical precipitation. The entire procedure is depicted in the provided Figure 7a.^[103]^ To mitigate the detrimental effects of high‐concentration H_2_SO_4_, researchers developed a high‐temperature activation process to stabilize the LFP olivine structures, followed by low‐concentration acid leaching for metal recovery. This effective method efficiently removes impurities and oxidizes the LFP, resulting in a successful and environmentally friendly battery recycling process.^[104]^ The process achieves high leaching rates of Li, Fe, and P, with approximately 85.56 % Li and 99.58 % Fe being successfully recovered as Li_3_PO_4_ and FePO_4_, offering a simple, efficient, and industrially feasible approach for battery recycling.

Indeed, besides sulfuric acid, other acids can be used to recycle LiFePO_4_ batteries. Researchers have explored leaching using other acids, such as phosphoric acid (H_3_PO_4_). In a moderate approach, LFP has dissolved in 0.88 M H_3_PO_4_ at temperatures of 80 °C, followed by filtration to eliminate undissolved components. Subsequently, the resulting solution underwent reflux to yield FePO_4_.2H_2_O precipitate. After thorough washing with distilled water and ethanol, the residue was dried at 80 °C, achieving a notable leaching efficiency of Li recovery of 99 %. Also, nearly all the iron is formed into FePO_4_.2H_2_O residue.^[105]^

Inorganic acids are preferred in hydrometallurgical processes for cost‐effectiveness and rapid leaching rate in extracting Li from LFP batteries. However, wastewater management and toxicity concerns must be addressed. Optimization should focus on minimizing acid concentration, reducing chemical consumption, and enhancing Li product purity and reuse value, considering the low economic value of LFP cathode material.

##### 3.2.2.2 Organic Acid Leaching

Due to the potential risks and environmental impacts associated with using inorganic acids, mild organic acids such as Oxalic acid, citric acid, aspartic acid, acetic acid, and formic acid are used as leaching reagents instead of inorganic acids. For example, Formic acid has been employed as the solvent and H_2_O_2_ as an oxidant in a selective leaching process for Li recovery from LiFePO_4_ spent.^[106]^ Figure 7b depicts the developed process for recovering lithium phosphate from the spent cathode powder of LiFePO_4_ batteries. The resulting product, Li_3_PO_4_, with a purity greater than 99 %, was precipitated in situ by directly adding a saturated solution of Na_3_PO_4_. When oxalic acid leaches the LFP material, it does not promote Fe^2+^ oxidation but precipitates FeC_2_O_4_. Conversely, other potential products such as Li_2_C_2_O_4_, Li_3_PO_4_, and Fe_3_(PO_4_)_2_ demonstrate solubility in acidic environments. The proposed reaction can be represented as follows^[85]^:

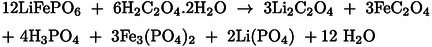




The researchers attained an impressive Li leaching efficiency of 98 % by employing 0.3 M oxalic acid at 80 °C for 60 min, with a solid/liquid ratio of 60 g/L. Using oxalic acid as a leaching agent, it can efficiently precipitate approximately 92 % of the iron content as FeC_2_O_4_.2H_2_O from LiFePO_4_.^[107]^ Another advantage of using oxalic acid as a leaching agent is that it does not generate any secondary pollutants, making it environmentally friendly and efficient.^[108]^ An eco‐friendly and cost‐effective process was developed using acetic acid as the leaching agent to selectively recover valuable metals from spent LiFePO_4_ batteries. With optimized parameters, lithium leaching achieved an efficiency of over 95.05 % and a selectivity of approximately 94.08 %. The resulting Li_2_CO_3_ had a high purity of 99.95 wt %, meeting battery‐grade standards. The process is economically viable and incorporates green chemistry principles, offering a sustainable solution for recycling spent LiFePO_4_ batteries.^[109]^

Generally, lithium may be selectively extracted using organic acids, but large‐scale recycling is not practical due to their cost. A thorough investigation of risk‐free alternatives to organic acid leachates is crucial in creating an effective, financially feasible procedure.

##### 3.2.2.3 Oxidation Leaching

Oxidation leaching is a hydrometallurgical process involving oxidizing agents to dissolve metals from ores or materials, allowing for metal recovery and extraction. For instance, sodium persulfate (Na_2_S_2_O_8_) can also be used to efficiently oxidize LiFePO_4_ to FePO_4_ and cause lithium extraction from the cathode without acid or alkali.^[110]^ It is shown that Na_2_S_2_O_8_ as an oxidant can facilitate the recycling of lithium from used LiFePO_4_ batteries. This method allows for efficient leaching of over 99 % lithium from the cathode without the use of acid or alkali. The process maintains the olivine crystal structure of the raw material, as shown in Figure 7c, and the resulting Li_2_CO_3_ product is of high purity (>99 %).

In addition to sodium persulfate, another used and effective oxidant for handling spent LiFePO_4_ batteries is H_2_O_2_. It acts as a powerful oxidant, breaking down into oxygen and water without introducing any impurities.^[111]^ However, the considerable costs and expenses associated with treating secondary waste present practical and feasibility challenges when considering the implementation of these methods on an industrial scale. To address these issues, another study investigates air oxidation‐water leaching as a cheap and eco‐friendly alternative for selectively extracting lithium from the spent LFP cathode material. The research achieves high leaching efficiency of lithium and effective separation of lithium and iron, showcasing a clean and efficient technology for recycling spent LFP batteries, aligning with the principles of green chemistry.^[112]^

##### 3.2.2.4 Bioleaching

Metal extraction from LIBs through bioleaching is an environmentally friendly process. It operates under ambient conditions, requiring less energy, resulting in fewer greenhouse gas emissions, lower operational costs, and reduced contamination and processing hazards compared to conventional pyrometallurgical and hydrometallurgical methods.^[113]^ Currently, a study successfully used Thiobacillus thioparus bacterium for efficient and eco‐friendly leaching of lithium from LFP battery cathodes, achieving high recovery rates of 65–98 %. The recovered lithium was transformed into Li_3_PO_4_ and reused as a culture medium, offering a sustainable solution for selectively reclaiming critical metals in battery production. Future research should focus on reducing reaction time, enhancing metal recovery at higher density, and exploring different bacterial strains to minimize acid consumption and waste treatment challenges.^[114]^

Although the bioleaching method is energy‐efficient and environmentally friendly, it has not been widely adopted due to problems with long cultivation times, slow process kinetics, low solid‐to‐liquid ratios, and metal toxicity. These issues call for advancements in metabolite production, microorganism metal tolerance, nutrient cultivation, and process kinetics.^[115]^ Table 2 displays the research findings on different leaching agents, optimal conditions, and the efficacy of Li leaching.

**Table 2:** Metal recovery from spent LFP batteries using the hydrometallurgy method.

Leaching system	Solvent	Leaching Condition			Resultant (Recovered Li)	Resultant (Recovered Fe)	Efficiency (%)		Ref
		Solvent conc. (M)	Temp (°C), Time (min)	Additive			Li	Fe	
**Inorganic acid**	H_2_SO_4_	2.5	60, 240	‐	Li_2_CO_3_	FePO_4_	97.2	98.5	^[102]^
	H_2_SO_4_	2.0	70, 120	‐	Li_3_PO_4_	FePO_4_	97.7	93.3	^[116]^
	H_2_SO_4_	0.3	60, 120	H_2_O_2_ (2.2 vol%)	Li_3_PO_4_	FePO_4_	96.9	0.03	^[101]^
	H_3_PO_4_	0.8	40, 40	H_2_O_2_ (4 vol%)	Li_3_PO_4_	FePO_4_	97.6	1.12	^[117]^
	HCl	20 wt %	60, 120	H_2_O_2_ (20 wt %)	Li_3_PO_4_	FeCl_3_	92.2	91.7	^[118]^
**Organic acid**	Oxalic acid	0.3	80, 60	‐	Li_2_C_2_O_4_	FeC_2_O_4_	98	92	^[107]^
	Acetic acid	0.8	50, 60	H_2_O_2_ (6 vol%)	Li_2_CO_3_	FePO_4_	95.1	94.1	^[109]^
	Formic acid	1	30, 30	H_2_O_2_ (5 vol%)	Li_3_PO_4_	FePO_4_	99.5	0.5	^[106]^
	Methyl Sulfonic	4	25, 90	H_2_O_2_ (18 %)	Li^+^	Fe^3+^	94	95	^[119]^
**Oxidant**	Na_2_S_2_O_8_	‐	25, 20	‐	Li_2_CO_3_	FePO_4_	>99	0.05	^[110]^
	H_2_O_2_	2.7	25, 240	‐	Li_2_CO_3_	FePO_4_	95.4	‐	^[120]^
	(NH_4_)_2_S_2_O_8_	‐	40, 60	‐	Li_2_SO_4_	FePO_4_	99	‐	^[121]^
	Fe_2_(SO_4_)_3_, NaCl	‐	60, 30	H_2_O_2_ (0.6 mol/g)	Li_2_CO_3_	FePO_4_	96.5	0.1	^[122]^
	air	600 mL/min	25, 300	‐	Li_2_CO_3_	FePO_4_	99.3	0.02	^[112]^
**Bioleaching**	T.thioparus, bacteria	‐	700, 30–60 days	‐	Li_3_PO_4_	FePO_4_	98	‐	^[114]^
	Lemon Juice	100 % juice	25, 60	H_2_O_2_ (6 vol%)	Li_2_CO_3_	FePO_4_	94.8	4.05	^[123]^

### 3.3 Direct Regeneration

**Figure 8:**
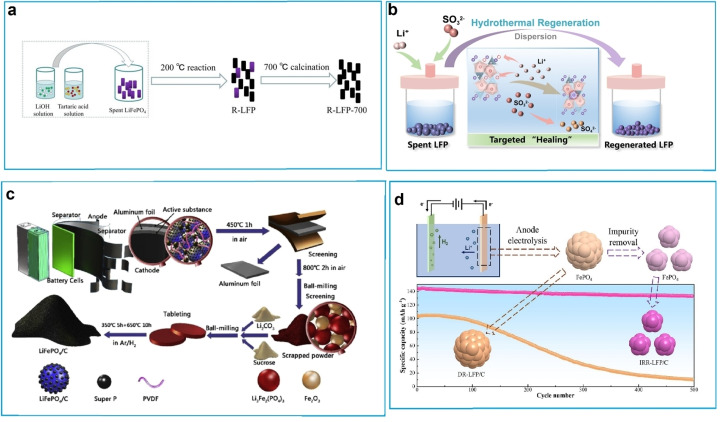
(a) Regeneration of spent LFP cathode. Copyright 2022 Elsevier. Reproduced with permission from reference,^[129]^ (b) Schematic illustration of the hydrothermal regeneration mechanism and particle size distribution of spent LFP and regenerated LFP. Copyright 2023 Elsevier. Reproduced with permission from reference,^[130]^ (c) the process of resynthesizing LiFePO_4_/C material. Copyright 2019 Elsevier. Reproduced with permission from reference,^[137]^ and (d) schematic description of Li recovery by electrochemical techniques. Copyright 2023 Elsevier. Reproduced with permission from reference.^[140]^

Direct regeneration is a non‐destructive method for recycling spent LFP cathodes, restoring their composition and structure without damage. It substantially cuts recovery costs versus traditional methods and surpasses other recycling technologies in energy efficiency, emissions, and revenues. It demonstrates technical superiority and promises widespread adoption.[^124^, ^125^] Various methods have been documented for the direct regeneration of depleted LFP cathodes. These methods include hydrothermal relithiation, molten salt repair, solid‐state sintering, and electrochemical treatment. Hydrothermal relithiation is a process used to reinsert lithium ions into spent lithium‐ion battery cathodes. Low‐temperature hydrothermal relithiation is a specific method that achieves this at temperatures at or below 100 °C, with the help of redox mediators like green additives to improve the process efficiency.^[126]^ Xu et al. introduced a clean hydrothermal relithiation process, involving combining low‐temperature aqueous solution relithiation with rapid post‐annealing. Through this method, they successfully demonstrated the direct regeneration to heal the structural defects of the spent LiFePO_4_ cathode.^[127]^ A one‐step hydrothermal method was employed to regenerate spent LiFePO_4_ cathode material directly, utilizing Hydrazine as an electron donor and reductant, enhancing electrochemical performance.^[128]^ A green hydrothermal technique is proposed for the direct regeneration of spent LiFePO_4_. The study investigates the effects of hydrothermal conditions on product performance, finding that controlling these conditions facilitates lithium supplementation and enhances electrochemical performance. Under 200 °C for 3 h (Figure 8a), the regenerated LFP shows optimal electrochemical performance, demonstrating the potential of this green and efficient method for lithium‐rich lithium‐based products.^[129]^ Yang et al. present a green hydrothermal relithiation technique using Li_2_SO_4_ solution as the lithium source and Na_2_SO_3_ as a reductant to directly regenerate spent LFP. The impacts of hydrothermal temperature, Li concentration, and reductant dose during LFP regeneration are carefully investigated in this work.^[130]^ Following hydrothermal regeneration, the particle size distribution (Figure 8b) exhibited a narrow and high peak attributed to the dispersion of LFP particles in the liquid phase.^[130]^

Molten salt offers superior ionic concentration and diffusion rates compared to aqueous solutions. Li‐based eutectic molten salts serve as both lithium sources and liquid mediums for reacting with transition metal precursors, facilitating the synthesis of high‐performance active materials under ambient pressure.^[131]^ Nitrate molten salt systems are suitable for low‐temperature lithium supplementation regeneration with layered cathode materials such as LCO and NCM. However, this system is not appropriate for regenerating LFP because nitrate salts, being highly oxidative, tend to oxidize Fe^2+^ to Fe^3+^.^[132]^ To address this issue, low‐temperature lithium nitrate molten salt along with a carbon reductive environment. By increasing the temperature to 300 °C, the melted lithium nitrate ensures close contact between lithium ions and the surface of spent LFP particles, compensating for lost lithium. This approach effectively capitalizes on the low melting point benefit of nitrate.^[133]^ Ji et al. used a multifunctional organic lithium salt (3,4‐dihydroxybenzonitrile dilithium) to restore the spent LiFePO_4_ cathode by direct regeneration.^[134]^ This interaction helps fill vacancies and creates a reducing environment, preventing the formation of Fe(III) phases. Furthermore, when the salt undergoes pyrolysis, it forms an amorphous conductive carbon layer. This layer improves the transfer of lithium ions and electrons, thereby enhancing the performance of the cathode. A rapid ammonium sulfate (NH_4_)_2_SO_4_ salt is proposed for extracting lithium and recycling FePO_4_ from commercial LiFePO_4_ cathode materials. Compared with the traditional hydrometallurgy recovery process, this method achieves fast delithiation of LiFePO_4_ within 5 min at 300 °C and converts LiFePO_4_ to FePO_4_ in an air atmosphere.^[135]^ As for regenerating methods, solid‐state sintering stands out as a universal technique and has proven to be effective in restoring the capacity of degraded cathodes.^[136]^ For example, a green solid route is introduced to resynthesize LiFePO_4_/C materials from spent lithium‐ion batteries, to recycle valuable resources and reduce environmental pollution.^[137]^ As illustrated in Figure 8c, the cathodes were crushed, dried, and heated to remove the binder and separate the cathode powder. Subsequent processes involved sintering, mixing with scrap powder containing sucrose and Li_2_CO_3_, pressing into tablets, and heating to resynthesize the LiFePO_4_/C materials with varying Li_2_CO_3_ addition amounts.^[137]^ A solid‐state method is currently reported for regenerating spent LiFePO_4_, involving powder homogenization followed by thoroughly mixing a lithium source and a carbon source via spray drying. Subsequently, carbon‐coated lithium iron phosphate is regenerated using a high‐temperature solid‐phase method. The regenerated LiFePO_4_@C demonstrates restored lattice structure, uniform surface carbon coating, and excellent electrochemical properties.^[138]^ For direct recycling, scrap cathode materials with a pristine structure can be regenerated through electrochemical processes. A proposed relithiation method utilizes a three‐electrode system involving the intercalation of lithium ions into scrapped LFP within an aqueous solution system. This process employs an H‐type electrolytic bath configuration with an anion‐exchange membrane, a zinc plate as the anode, scrapped LFP suspension as the cathode, and a lithium salt aqueous solution as the electrolyte, ultimately leading to LFP regeneration through a discharging process.^[139]^ In addition, an electrochemical approach is employed for the selective recovery of lithium from spent LiFePO_4_ through anodic electrolysis (Figure 8 d). Optimized conditions result in high leaching rates for Li (96.31 %), while minimizing leaching of Fe (0.06 %) and P (0.62 %), with an exceptional Li/Fe selectivity exceeding 99.9 %.^[140]^ The main reactions during electrolysis are as follows:

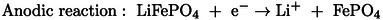




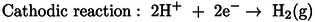




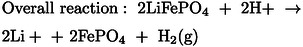




### 3.4 New Progress in the Recycling of LiFePO_4_

When discussing metal separation units, eco‐friendly and sustainable recovery methods are consistently considered to have higher research value and significance. As a response, researchers have been exploring innovative approaches for the green recovery and reuse of LFP cathode materials. These methods aim to achieve efficient metal reclamation while promoting environmental sustainability and cost‐effectiveness. This section explores some strategies, highlighting their potential to shape a more eco‐friendly and economically viable future for LFP recycling.

**Figure 9:**
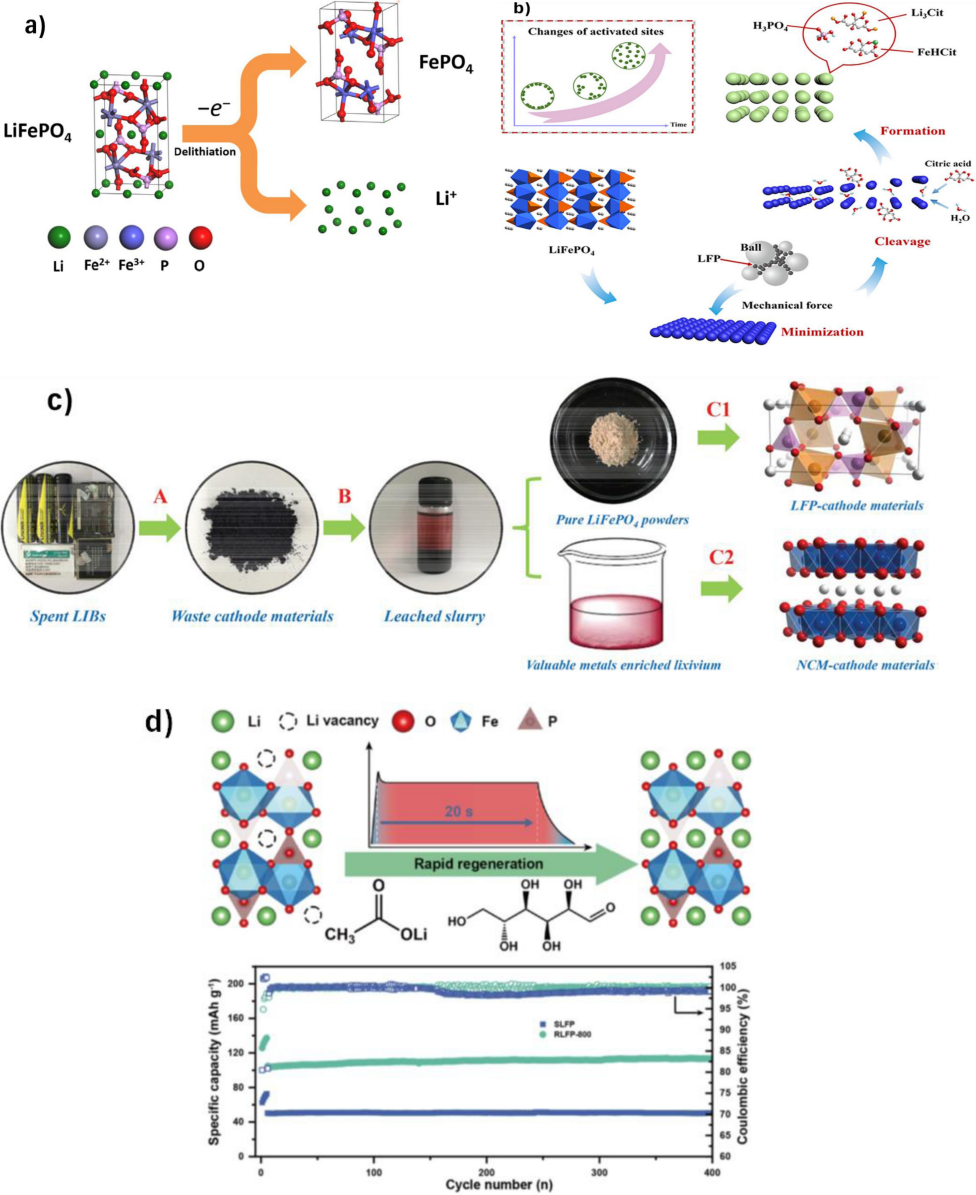
(a) Schematic illustration of the leaching mechanism for extraction of Li^+^. Copyright 2020 Elsevier. Reproduced with permission from reference,^[142]^ (b) mechanism of the mechanochemical treatment of spent LiFePO_4_. Copyright 2019 Elsevier. Reproduced with permission from reference,^[145]^ (c) the simultaneous recovery of valuable metals and iron from mixed types of spent LIBs. Copyright 2019 Royal Society of Chemistry. Reproduced with permission from reference,^[147]^ and (d) diagram for the rapid regeneration of spent LFP cathode materials. Copyright 2023 Elsevier. Reproduced with permission from reference.^[152]^

Researchers established a novel in‐situ recycling process for spent LFP batteries using ultrasound‐assisted Fenton reaction and direct cathode material regeneration. In this innovative approach, an ultrasound‐assisted Fenton reaction was utilized to selectively remove PVDF binders, allowing for the recovery of LiFePO_4_ cathode materials from current collectors. They effectively separated LiFePO_4_ cathode materials from Al foils with a high liberation rate. Under optimized conditions, about 97 % of coating materials detached with minimal lithium loss. The process involved hydroxyl radicals (⋅OH) degrading PVDF binders, facilitating cathode material separation. The exfoliated cathode materials were directly regenerated and reused, exhibiting electrochemical performance comparable to commercial LiFePO_4_.^[141]^ A straightforward and eco‐friendly approach was employed to recycle spent LiFePO4 batteries. The method involved combining the charging mechanism utilized in LiFePO_4_ batteries with a slurry electrolysis process. Researchers could efficiently separate Li and FePO4 by anionic membrane without requiring additional chemicals. chemicals. In aqueous rechargeable LIBs, the charging mechanism of the LFP cathode material involves oxidizing LFP to FePO_4_ at a positive potential. During this process, Li^+^ ions are released into the aqueous electrolyte and later recovered. Inspired by this mechanism, researchers explored electrochemical Li extraction as a potential method for recovering Li from LFP cathode materials. Utilizing electrochemical techniques to extract Li ions selectively presents a promising and sustainable route for Li recycling in LIBs.^[142]^ During the slurry electrolysis process, lithium ions are easily extracted and leached from the olivine structure of LiFePO_4_, compensating for the oxidation from Fe^2+^ to Fe^3+^ and leaving FePO_4_ in the leaching residue (Figure 9a).

Mechanochemical technology offers a promising approach to recycling metals from liquid‐liquid‐phase LIBs through the utilization of mechanical forces, such as milling or grinding, to initiate chemical reactions that facilitate the separation and extraction of valuable components from the battery electrodes.^[143]^ An economically viable acid‐free mechanochemical process has been developed for the selective recycling of spent LFP batteries. Researchers selectively extract Li from LFP cathode materials while preserving FePO_4_, using sodium citrate as a co‐milling agent. The method achieves 98.9 % selectivity and is economically viable, offering a promising solution for efficient and environmentally friendly recycling.^[144]^ Also, a mechanochemical process was employed by co‐grinding spent LiFePO_4_ with a cost‐effective citric acid agent in a ball mill. After grinding, the mixture is dissolved in deionized water and filtered. By introducing H_2_O_2_, lithium extraction reaches an impressive 99.35 %, showcasing highly effective recovery. In contrast, iron extraction is much lower at 3.86 %, indicating selective lithium recovery over iron and using H_2_O instead of H_2_O_2_ leads to slightly lower extraction efficiencies for both lithium (97.82 %) and iron (95.62 %) under optimal conditions. The reaction proceeds through a minimization‐cleavage‐recombination process, as illustrated in. Figure 9b. This figure illustrates how the mechanochemical treatment of spent LiFePO_4_ results in the effective extraction of metals, demonstrating the importance of mechanical force and chemical reactions in the recycling process.^[145]^

Numerous studies have focused on recycling individual spent cathode materials, deemed unsuitable for practical applications. Recycled mixed cathode materials, including LFP, LMO, LNCM, and LCO, creating a new process is noteworthy. Zou et al. proposed a recovery process for mixed cathode materials, such as LCO, LMO, LNCM, and LFP, which involves leaching, precipitation, and synthesis steps to obtain purified materials for cathode synthesis. Analysis results demonstrate nearly 100 % recovery of Ni, Mn, and Co, while approximately 80 % of Li is recycled as Li_2_CO_3_.^[146]^ A closed‐loop process is proposed to simultaneously recover various metals from complex waste streams of spent LIBs. This study explores recycling valuable metals and iron from mixed types of spent LIBs. It employs selective leaching and solid‐phase fabrication to recover them as new cathode materials, specifically LiFePO_4_ and LNMC (Figure 9c).^[147]^ The recycling of mixed spent LIBs often requires excessive acid consumption and the use of multiple redox additives. A salt leaching method was implemented to address this challenge, utilizing NH_4_Fe(SO_4_)_2_ as a redox intermediate for synergistic recovery of valuable metals from mixed spent ternary NCM and LFP batteries. This approach achieves high efficiency and selectivity, preserving the environment through precipitate recovery and regeneration of new materials.^[148]^ A synergistic redox strategy has been introduced to recover all elements from mixed systems spent LFP and NCM batteries, utilizing thermodynamic calculations to identify self‐promoting reactions. With a less acidic environment, this method eliminated the need for additional agents and produced over 100 % leaching rates for manganese, nickel, cobalt, and lithium.^[149]^ As lithium‐ion batteries degrade, their capacity decreases due to irreversible lithium‐ion consumption, increased internal resistance, and structural damage, reducing lithium in the cathode and increasing lithium in the anode. Therefore, a graphite prelithiation strategy can directly reuse residual lithium from spent graphite anodes to regenerate degraded LiFePO_4_.^[150]^ A graphite prelithiation technique was presented to restore wasted LFP and offset Li^+^ ion loss.^[151]^ However, due to the complex pretreatment required in this process, there is a need for a faster method to regenerate spent LFP to save energy and costs. An ultrafast heating method is proposed to regenerate spent LFP within seconds, aiming to achieve low energy consumption and high efficiency in regeneration.^[152]^ This method, as illustrated in Figure 9 d, demonstrates the temperature variations during ultrafast calcination. This process involves exposing the regenerated LiFePO_4_ cathodes to temperatures of 700 °C, 800 °C, and 900 °C for a duration of 20 s each. It visually outlines the key steps of the high‐temperature shock strategy for cathode regeneration.

## 4 Analyzing the Operational Capacity for Recycling LFP Batteries

### 4.1 Market Share

The global lithium‐ion battery market, valued at USD 54.4 billion in 2023, is set to undergo robust growth, with a projected compound annual growth rate of 20.3 % from 2024–2030. This surge is primarily driven by the automotive sector, with a growing demand for lithium‐ion batteries due to their cost‐effectiveness. Researchers highlight the significance of energy storage as essential for human survival, as projections indicate a demand for 2800 GWh of batteries by 2030 and exceeding 9000 GWh by 2050. As nations shift towards electric vehicles, surpassing 50 million globally by 2030, the necessity for energy storage solutions becomes evident, especially with replacing traditional fuel vehicles.^[153]^

**Figure 10:**
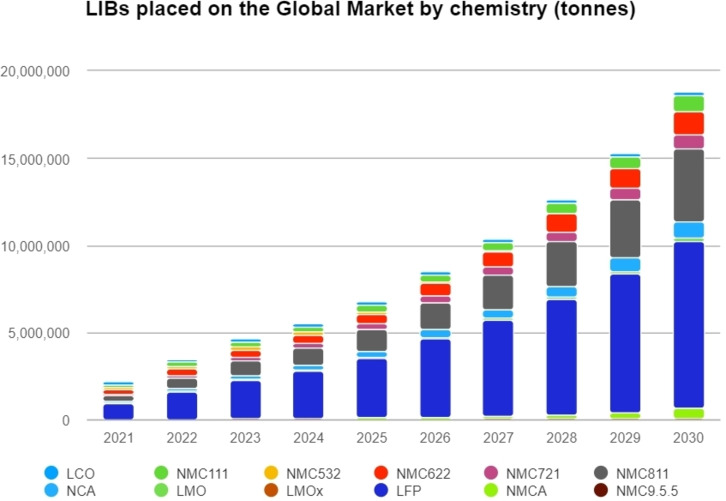
LIBs on the global market by chemistries. Copyright CES Online 2024. Reproduced with permission from reference,^[157]^ (paid subscription).

LIBs serve primarily in consumer electronics, electromobility, and stationary energy storage. Mobile phones in consumer electronics rely on LCO (87 %) and NMC batteries (13 %) due to their higher energy density than LFP batteries. LFP and LMO batteries excel over LCO in cost and safety, making them preferable choices. LFP batteries are widely used in EVs and electrical storage systems due to their affordability, safety, and durability. NCM batteries, valued for their high energy density, are also standard in EVs, while NCA batteries encounter production challenges. In contrast, LMO batteries have limited market share due to their lower energy density and temperature performance, allowing LFP and NCM batteries to dominate the market.[^154^, ^155^] This paper focuses on LIBs utilizing LFP as the cathode active material. With 32,400 tonnes shipped in 2015, China, the world's largest producer and consumer of LiFePO_4_, held 65 % of the market share worldwide.^[156]^ The forecast indicates that the global LFP cathode active materials market will grow significantly from 900,000 tonnes in 2021–9,500,000 tonnes in 2030. This growth is accompanied by an increasing relative market share, rising from 42 %–51 %. The market share of various LIBs is illustrated in Figure 10, with forecasts extending up to 2030.

### 4.2 Collection and Sorting

The public's understanding of LIB management has grown over recent decades as governments enact more battery collection and disposal regulations, recognizing the need for an extensive collection infrastructure. Just North America, Asia, and Europe engage in LIB recycling. The EU has developed various policies and strategies to advance battery technologies in alignment with decarbonization, energy, raw materials, the circular economy, and innovation. With a rising demand for batteries, the EU stresses the importance of boosting production capacity and reducing dependency on non‐European suppliers, focusing on achieving climate targets, enhancing industrial competitiveness, and ensuring environmental sustainability. This is facilitated through initiatives like the Strategic Action Plan for Batteries, which aims to secure raw materials, advance technology, bolster battery production, cultivate skilled human resources, and advocate for sustainable battery practices.^[158]^ In Europe, regulations for battery management are governed by directives such as Directive 2006/66/EC, Directive 2012/19/EU, and Directive 2000/53/EC, which cover waste batteries, waste electrical and electronic equipment, and end‐of‐life vehicles, respectively. These directives aim to ensure proper collection, recycling, and disposal of various types of batteries, including those used in portable devices, vehicles, and industrial applications. Despite efforts to meet recycling targets, challenges remain in enhancing collection efficiency and reducing landfilling or incineration of spent LIBs.[^159^, ^160^, ^161^]

Meanwhile, federal regulations under the Universal Waste Regulations in the USA provide a framework for managing hazardous waste, but LIBs are not considered hazardous and are largely excluded. Some states have additional regulations focusing on battery recycling, but there are no defined recycling targets or penalties for noncompliance. Similarly, in China, regulations are being developed to manage waste traction batteries, driven by initiatives like the “Energy‐saving and New Energy Vehicles Industry Development Program,” aiming to establish recycling networks and advanced processes for battery reuse.[^154^, ^156^, ^158^] Collecting batteries, including LIBs, Pb‐acid batteries, NiCd batteries, and NiMH batteries, typically involves placing them in designated containers at collection centers or sorting them directly at treatment plants, often performed manually. Once collected, LIBs are processed collectively as mixed feed, although some industrial facilities may sort them by sub‐chemistry, such as LCO, LFP, or LMO. However, detailed information about this sorting process is often unavailable.^[156]^

### 4.3 Recycling Market

**Figure 11:**
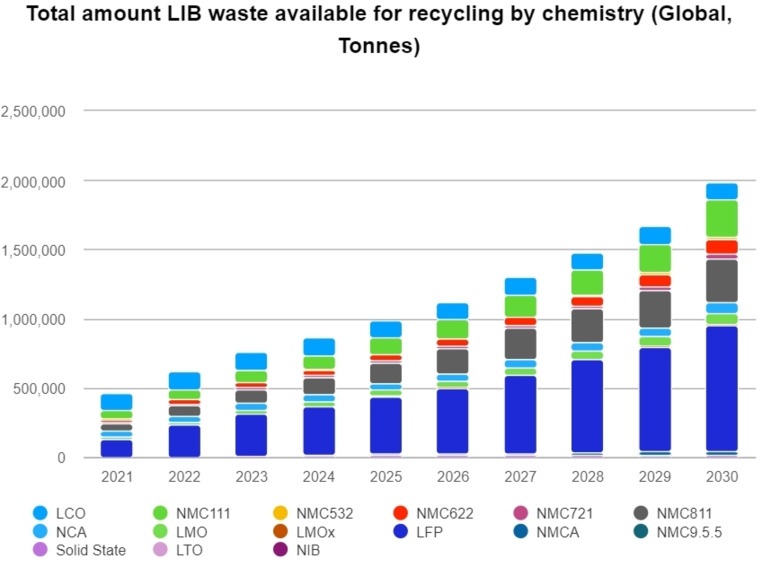
Total amount of LIB waste available for recycling by chemistry. Copyright CES Online 2024. Reproduced with permission from reference,^[157]^ (paid subscription).

The number of batteries available for recycling by 2030 is not expected to grow at the same rate as the new battery market, mainly due to longer battery lifetimes and increased reuse. While the battery market is predicted to grow annually by 27.7 % between 2020 and 2030, the recycling rate is forecasted to decrease each year. Changes in the recycling rate depend on factors such as battery production scrap volume and the reuse rate, which will be closely monitored and refined in models to improve accuracy. Additionally, regional dynamics, including battery production and trade, influence the recycling ratio in different markets. Key indicators for 2022 include monitoring battery production capacity, prices for batteries intended for reuse and recycling, and studies on production scrap and alternative scrap sources to enhance modeling accuracy Circular Energy Storage Research forecasts a significant rise in annual return flows of LFP batteries, projecting an increase from 230,000 tonnes in 2022 to about 900,000 tonnes by 2025, as depicted in Figure 11.^[157]^

**Figure 12:**
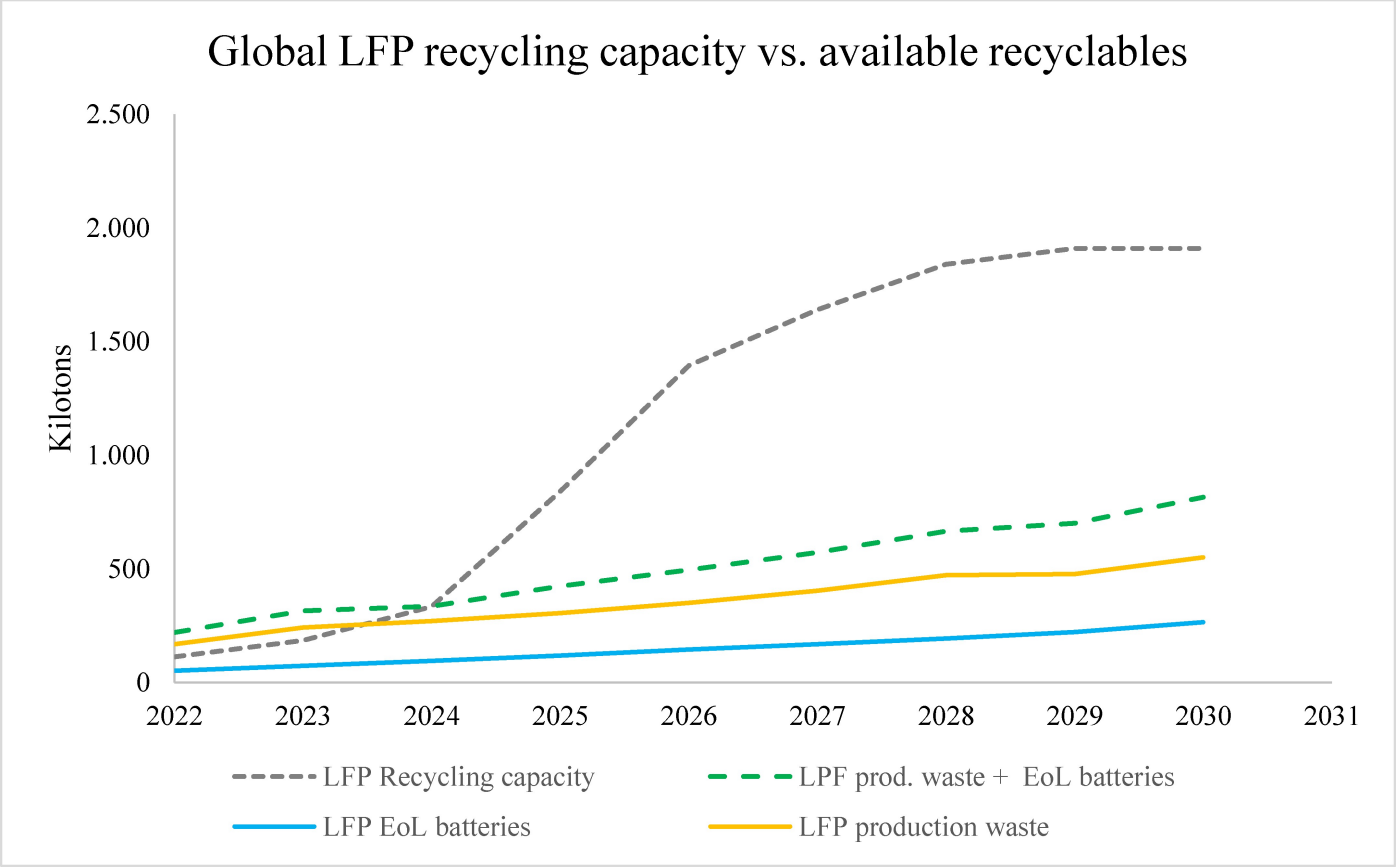
The expected development of LFP recycling capacities vs. recyclables available 2022–2030. Copyright CES Online 2024. Reproduced with permission from reference,^[157]^ paid subscription).

According to the estimation of Circular Energy Storage, the current industrial annual recycling capacity for LFP chemistry is about 200 kilotons, thus falling short of the production waste and EoL LFP batteries produced yearly.^[157]^ The situation is expected to change rapidly within a few years: recycling is expected to increase almost 10‐fold to 1,900 kilotons by 2030, while the amount of recyclables is estimated to be 815 kilotons, of which the actual EoL batteries are 265 kilotons (Figure 12). The majority of the recycling capacity increase is expected to occur in China, where most LFP production is concentrated^[163]^ and, thus, where the LFP production waste is born. A list of current industrial operators recycling LFP chemistry is gathered in Table 3.

**Table 3:** Operational or pilot lithium‐ion battery recycling plants having capacity/capability for recycling LFP chemistry. Data retrieved from reference.^[157]^

Company	Region	Recycling capacity 2023, LFP, tons	Recycling capacity 2023, all chemistries, tons	Products of the recycling plant (all the listed products, e. g. not only products from recycling LFP)	Recycling process phase	Source * Recycling capacity ‐ Products Other information: CES online
Austin Elements	United States	26	40	Chemical salts, lithium carbonate, lithium hydroxide	Material recovery	*‐ CES online https://www.circularenergystorage‐online.com/therecyclingmarket/globalrecyclers/austin‐elements/houston
Brunp	China		120 000	Aluminum metal, chemical salts, copper metal, lithium carbonate, precursors, cathode material	Pre‐processing & Material recovery	*‐CES online https://www.circularenergystorage‐online.com/therecyclingmarket/globalrecyclers/brunp/ningxiang Argus Media https://www.argusmedia.com/en/news/2280302‐chinas‐brunp‐builds‐yichang‐battery‐material‐complex
CALB	China	1000		Aluminum metal, copper metal, lithium carbonate, precursors	Pre‐processing & Material recovery	*‐CES online https://www.circularenergystorage‐online.com/therecyclingmarket/globalrecyclers/calb/changzhou
Chizhou Xian New material Technology	China		6 000 ^(1)^	Aluminum metal, copper metal, lithium carbonate, precursors	Pre‐processing & Material recovery	* Company webpage http://www.cnntech.cn/page/endchs.html ‐ CES online: https://www.circularenergystorage‐online.com/therecyclingmarket/globalrecyclers/chizhou‐xian‐new‐material‐technology/chizhou
Full Circle Lithium	North America	500		Lithium carbonate, Aluminum metal, Copper metal	Pre‐processing & Material recovery	*‐ CES online https://www.circularenergystorage‐online.com/therecyclingmarket/globalrecyclers/full‐circle‐lithium/nahunta
Ganzhou Longkai Technology	China		60 000	Aluminum metal, copper metal, lithium carbonate, precursors	Pre‐processing & Material recovery	*‐ CES online https://www.circularenergystorage‐online.com/therecyclingmarket/globalrecyclers/chizhou‐xian‐new‐material‐technology/chizhou
GEM	China	3 000	130 000	Precursors	Pre‐processing & Material recovery	*‐https://cleantechnica.com/2021/06/20/gem‐is‐a‐gem‐in‐the‐battery‐recycling‐industry‐that‐was‐inspired‐by‐a‐toothpaste‐experiment/ & CES online
Guangdong Weima New Materials	China	12 000		Lithium carbonate, aluminum metal, chemical salts, copper metal	Pre‐processing & Material recovery	‐*CES online
Guanghua Technology	China		50 000	Cobalt sulfate, nickel sulfate, manganese sulfate, iron phosphate, lithium iron phosphate, lithium manganese iron phosphate, lithium carbonate, and lithium carbonate concentrate	Pre‐processing & Material recovery	*CES online ‐ Company webpage https://www.ghtech.com/Eapplication/recovery_100000000297454.html
Hengchuang Ruineng Environmental Protection Technology	China	50 000		Lithium carbonate, aluminum metal, chemical salts, copper metal	Pre‐processing & Material recovery	*‐CES online
Hunan Wuchuang Recycling Technology	China	12 000		lithium carbonate, aluminum metal, chemical salts, copper metal	Pre‐processing & Material recovery	*‐CES online
Jiangxi Ganfeng Recycling Technology	China		70 000	anhydrous lithium chloride Battery grade lithium carbonate, lithium hydroxide and other products	Pre‐processing & Material recovery	* CES online ‐ https://www.ganfenglithium.com/pro3_detail_en/id/164.html
Kyburz	Switzerland	300		Lithium iron phosphate, copper, graphite, aluminum, plastics, polymer foil	Pre‐processing	*CES online https://www.circularenergystorage‐online.com/therecyclingmarket/globalrecyclers/kyburz/freienstein ‐https://kyburz‐switzerland.ch/en/battery‐recycling
(Lanzhou Jinchuan Resource Recycling Technology)	China		25 000		Pre‐processing & Material recovery	
Nantong Beixin New Energy Technology	China	20000		Lithium carbonate, aluminum metal, chemical salts, copper metal	Pre‐processing & Material recovery	‐*CES online
Ningxia Baichuan New Materials	China		15 000	Lithium carbonate, nickel sulfate, cobalt sulfate, manganese sulfate	Pre‐processing & Material recovery	*CES online ‐ company webpages http://www.bcchem.com/en/product/100.html
ReElement	United States	137		Chemical salts, lithium carbonate, lithium hydroxide	Material recovery	*CES online+https://www.reelementtech.com/
Roth International	Germany		9 000	Copper metal, Aluminum metal, Black mass	Pre‐processing	*‐ CES online https://www.circularenergystorage‐online.com/therecyclingmarket/globalrecyclers/roth‐international/wernberg‐k%C3%B6blitz
Young Poon	South Korea		2 000	Chemical salts, lithium carbonate	Pre‐processing & Material recovery	*‐ CES online https://www.circularenergystorage‐online.com/therecyclingmarket/globalrecyclers/young‐poong/seoul
Zhejiang New Era Zhongneng Cycle	China		50 000	Lithium carbonate, cobalt chloride, cobalt sulfate, nickel sulfate, manganese tetroxide,	Pre‐processing & Material recovery	*CES online ‐ Company webpage http://en.zhongnengrecycling.com/product/

(1): In CES online stated that only LFP accepted and thus the 6000 tonnes capacity to recycle would consist only LFP. In company webpages it is however stated: “This project can recycle 6000 tons of spent ternary cathode materials annually”.

## 5 Future Challenges and Perspectives

Numerous researchers have extensively investigated the recycling of spent LFP batteries. However, current methodologies face significant limitations, and much of the research is conducted in laboratory settings. Expediting the transition from experimental stages to large‐scale industrial implementation is imperative. Unlike NCM or LiCoO_2_ batteries, the recycled material from LFP cathodes lacks the value of precious metals. Consequently, the intricate and costly recovery process hampers the industrialization of lithium iron phosphate battery recycling efforts. The following briefly describes the challenges encountered in the recycling of spent LFP batteries: 1) LFP batteries have achieved a substantial market presence within LIBs, mainly due to the rise of electromobility and stationary energy storage. However, the prevalence of LFP in certain regions poses unique challenges for future recycling programs. 2) Current universal recycling techniques, generally suitable for layered oxides, are inadequate for LFP batteries. Mechanical processing is being tested on a small scale, while hydrometallurgical processes are still in the laboratory's experimental stage, complicating the recycling process. 3) Compared to layered oxides, LFP batteries contain fewer metals, which raises questions about the financial viability of their recycling. The solution is creating a closed‐loop system emphasizing the importance of refining costs over raw material prices, thereby making LFP recycling economically feasible. 4) The preliminary findings from the Life Cycle Assessment cast doubt on the presumed environmental advantages of recycling LFP cells. This underscores the imperative for a thorough examination encompassing economic and environmental considerations. Should profitability prove elusive, policy interventions such as implementing a producer responsibility principle akin to the EU model may be warranted. Collaborative endeavors in research and development must be undertaken to establish economically viable and environmentally sustainable practices for recycling LFP batteries. Alignment with sustainable resource management principles and the capacity to adjust to changing recycling issues are prerequisites for this 5) Future research and development efforts should strongly emphasize creating flexible and scalable procedures specific to LFP batteries. In‐depth evaluations of the process's effects on the environment and economy must be performed at every stage. By methodically addressing obstacles, scientists may identify crucial elements impacting expenses and environmental effects, opening the door for more successful LFP battery recycling programs.

## 6 Conclusions

Considerable attention has been drawn to LFP as a promising cathode material for lithium‐ion batteries, owing to its advantages over conventional materials such as Co and Ni in terms of toxicity and cost‐effectiveness. Despite its current commercial application, there is a pressing need for more economical production methods. This review explores various synthesis approaches for LFP powders and assesses techniques for recycling spent LFP batteries, underscoring the importance of recycling and advocating for lithium‐ion battery sustainability. While numerous researchers have delved into recycling spent LFP batteries, current methodologies encounter significant limitations, mainly due to experimental constraints in laboratory settings. Challenges, including the absence of precious metals in recycled LFP cathodes, impede industrialization efforts. Future research endeavors should prioritize the development of adaptable and scalable procedures tailored to LFP batteries, accompanied by comprehensive evaluations of their environmental and economic impacts, thus facilitating the advancement of more effective recycling initiatives.

## Conflict of Interests

The authors declare no conflict of interest.

## Biographical Information

*Hossein Rostami holds a Ph.D. in electrochemistry from the University of Mazandaran (2016) and is currently a university researcher at the University of Oulu, working in the Research Unit of Sustainable Chemistry. His research focuses on the synthesis of cathode materials for Li‐ion batteries, as well as on coating, doping, battery cell preparation, and various characterization techniques*.

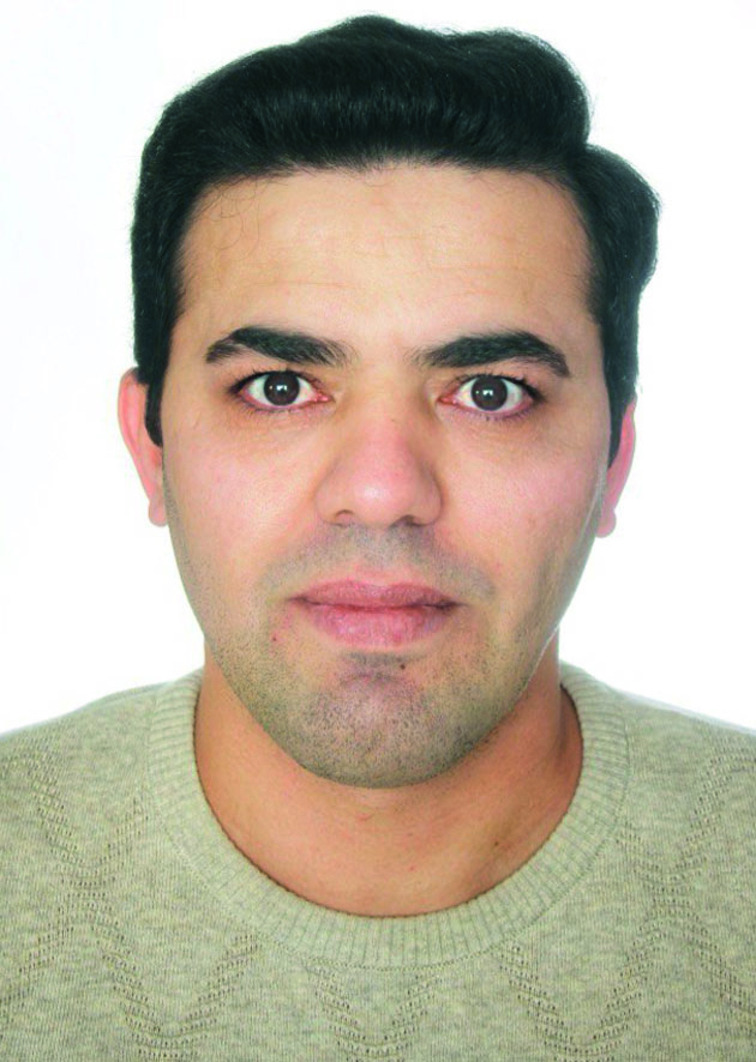

## Biographical Information

*Johanna Valio holds a Master of Social Sciences in sociology from the University of Joensuu (2003) and a Master of Science (Tech) in Chemical Engineering from Aalto University (2017), specializing in Functional Materials. Currently, Johanna Valio works as a researcher at the RoboAI research centre, part of Satakunta University of Applied Sciences, and as a principal investigator in AIST project (A novel AI‐based Spectroscopic Technique for recycling of battery materials). Her research interests include holistic understanding of battery recycling*.

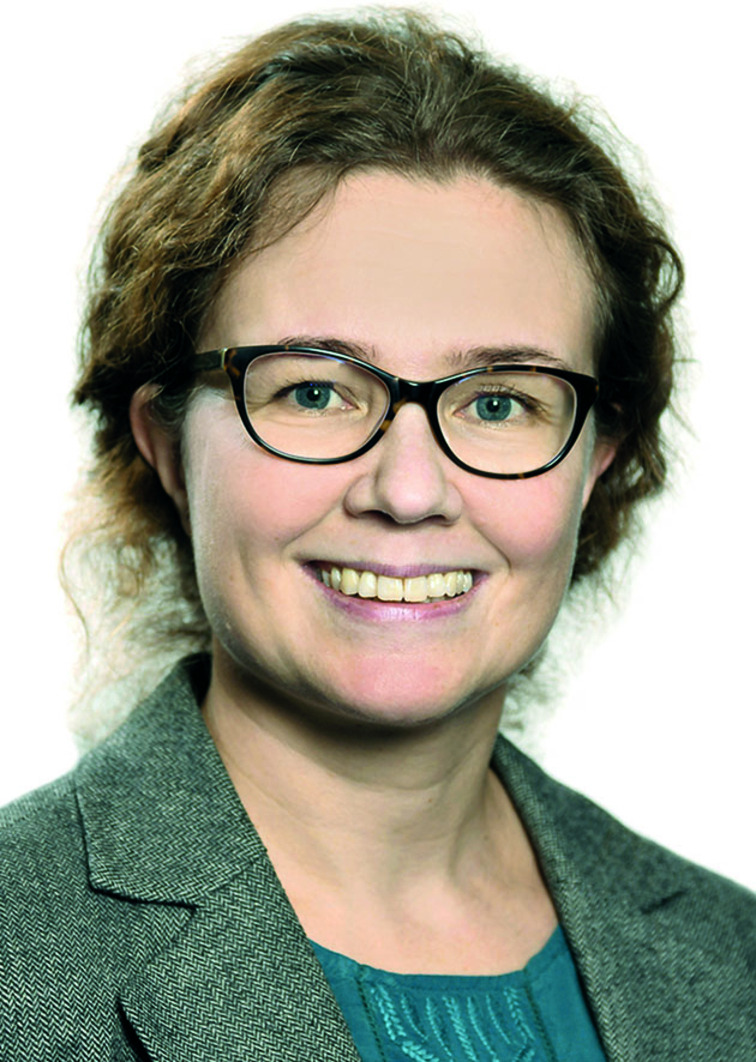

## Biographical Information

*Pekka Suominen, Doctor of Philosophy, Physics (2006 University of Jyväskylä), Head of Research in RoboAI research centre at Satakunta University of Applied Sciences (25 employers). His research interests are related to automation, robotics, artificial intelligence and their industrial applications, especially in laser‐induced breakdown spectroscopy and circular economy of technology metals and battery materials. He has 45 scientific publications and five patents. He has been an entrepreneur for 14 years and has founded several start‐up companies. Suominen has been principal investigator in 10 research projects*.

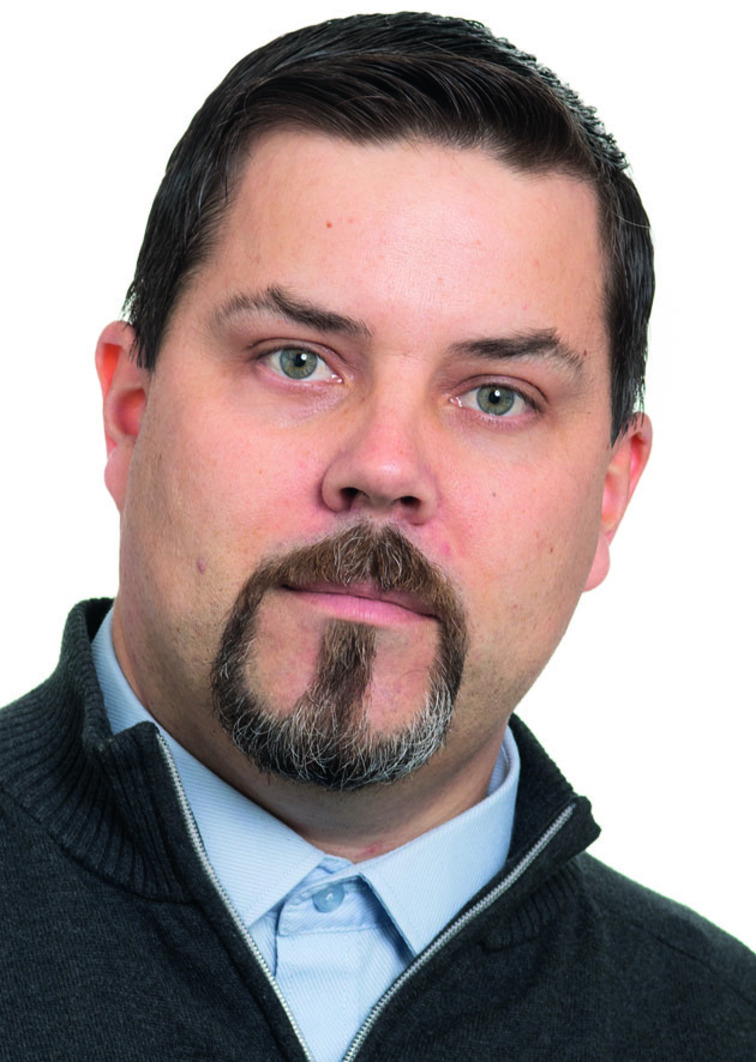

## Biographical Information

*Pekka Tynjälä, Doctor of Philosophy (University of Joensuu, 1998). He works as a university researcher at the University of Oulu in the Research Unit of Sustainable Chemistry. His research activities include hydrometallurgical processes such as co‐precipitation of lithium‐ion battery precursor materials and leaching of metallic materials in order to prepare battery‐grade metal sulfate solutions*.

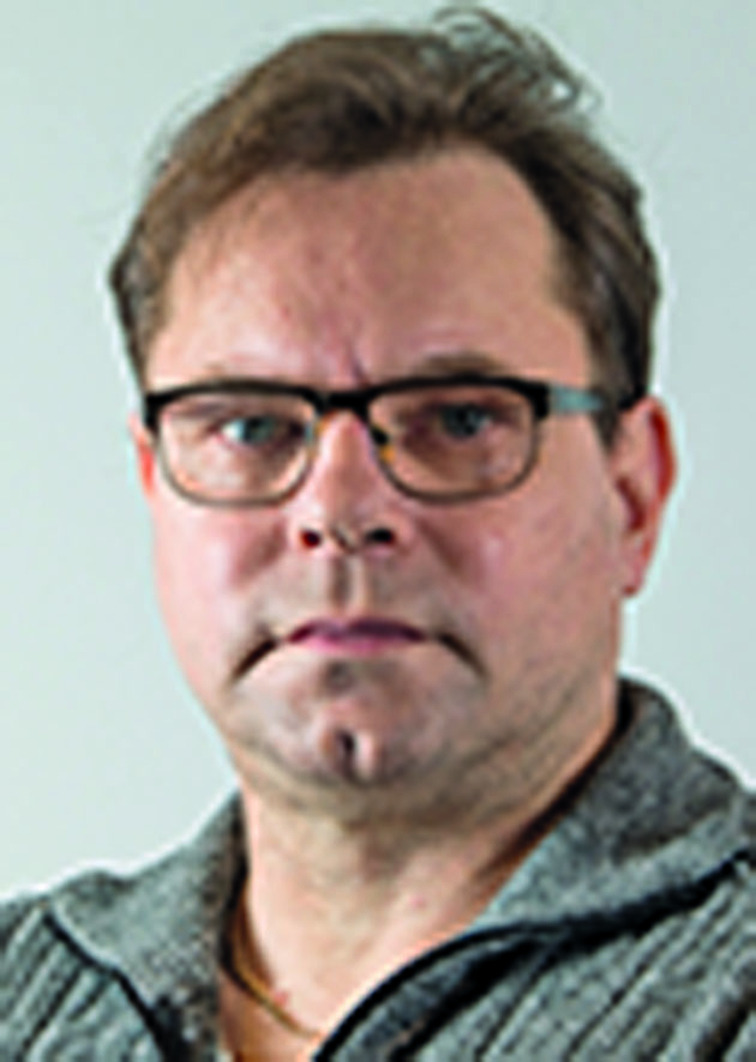

## Biographical Information

*SHORT CURRICULUM VITAE, Professor Ulla Lassi. Ulla Lassi, Doctor of Technology (2003), Professor at the University of Oulu in the Faculty of Technology (2006−). She is also the Head of Research Unit of Sustainable Chemistry (67 employers). Her research areas involve inorganic material chemistry in industrial applications, esp. catalysis and battery chemicals. She has 230 scientific publications and five patents. As a professor, she has supervised 30 PhD theses and 130 M.Sc theses. Lassi has been principal investigator of 40 research projects. She has several positions of trust. Lassi has also been awarded Tandem Industry Academy Professorship in battery chemistry for 2023–2025*.